# Visible light-mediated 1,2-difunctionalization reactions involving 4-cyanopyridines

**DOI:** 10.1039/d5ra09281g

**Published:** 2026-02-10

**Authors:** Pooya Raymand, Arman Farahani, Fatemeh Doraghi, Mohammad Hadi Edareh, Mehran Ghasemi, Mohammad Mahdavi

**Affiliations:** a Endocrinology and Metabolism Research Center, Endocrinology and Metabolism Clinical Sciences Institute, Tehran University of Medical Sciences Tehran Iran momahdavi@tums.ac.ir; b Center for Research of Endemic Parasites of Iran, Tehran University of Medical Sciences Tehran Iran; c Natural and Medical Sciences Research Center (NMSRC), University of Nizwa Nizwa 616 Sulanate of Oman

## Abstract

Pyridines are important heterocycles widely existing in natural products, pharmaceuticals, agrochemicals, and functional materials. Pyridines also serve as versatile synthetic building blocks for complex pyridines, as well as important ligands for transition metals. Over the past few years, 4-cyanopyridine has been successfully utilized for photocatalyzed 1,2-difunctionalization of alkenes. Hence, this review highlights multicomponent reactions involving 4-cyanopyridines and discusses their mechanistic insights.

## Abstract 2

Due to producing persistent pyridyl radicals, 4-cyanopyridine is widely used in the decyanative pyridylation reactions towards C4-functionalized pyridine derivatives. This review highlights 1,2-difunctionalization reactions involving 4-cyanopyridine.

## Graphical abstract

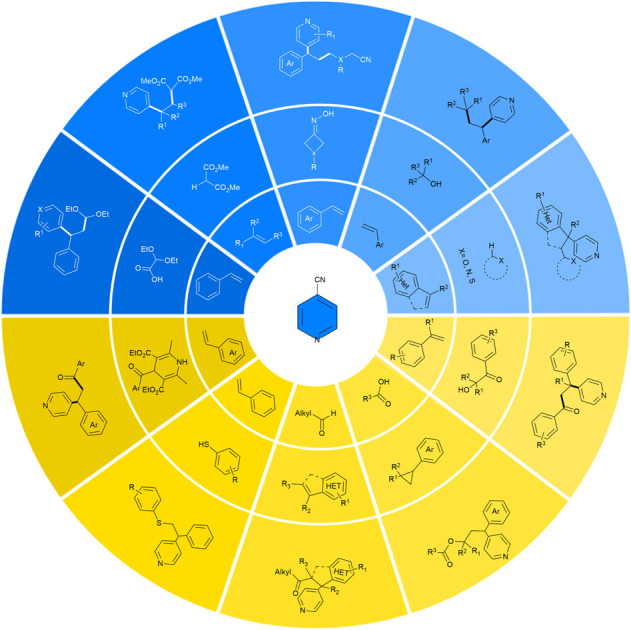

## 1. Introduction

**Fig. 1:**
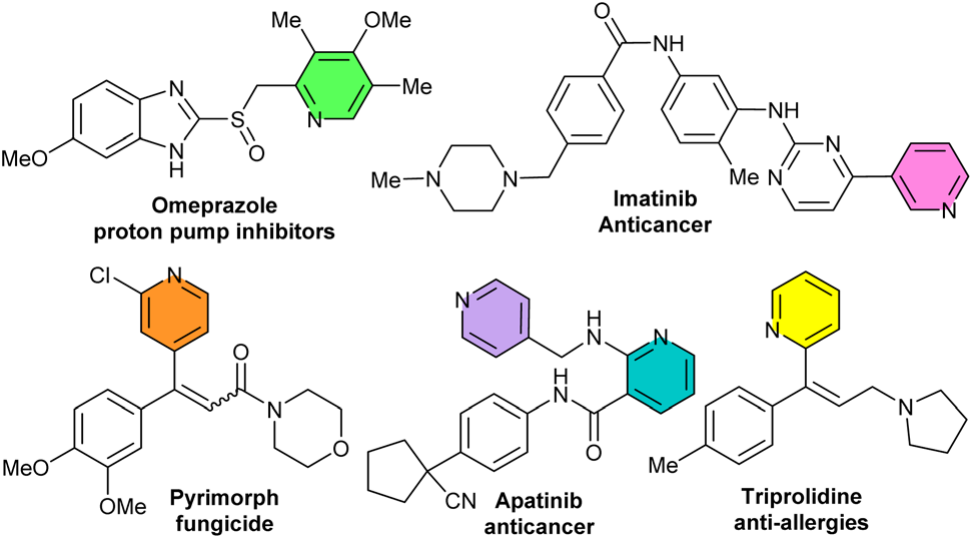
Representative biologically active molecules containing a pyridine core.

Due to its unique physical and chemical characteristics, pyridine is one of the most important structural motifs found in biologically active natural products,^1–3^ pharmaceuticals,^4–6^ agrochemicals,^7^ ligands,^8^ intermediates,^9,10^ functional materials,^11,12^ and so on. Pyridine-containing molecules are the most common heterocycles that exist in FDA-approved pharmaceuticals.^13^ Notably, top-selling drugs such as the proton pump inhibitor (Omeprazole),^14^ and targeted cancer therapy medications (Imatinib and Apatinib) include pyridine cores. Some drug molecules containing a pyridine motif are depicted in Fig. 1.

**Fig. 2:**
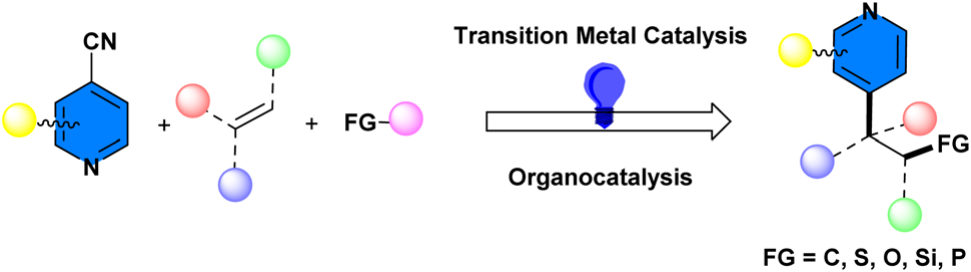
Photo-induced radical–radical coupling of 4-cyanopyridines with alkenes and other radical sources.

Commercially available 4-cyanopyridine has been extensively utilized for photocatalytic reductive pyridylations, especially decyanative pyridylation of alkenes,^15,16^ accompanied by β-functionalization, such as alkylation, alkoxylation, arylation, perfluoroalkylation, sulfonylation, silylation, phosphinoylation, *etc.* to give C4-functionalized pyridines. In such transformations, 4-cyanopyridine readily accepts an electron and produces a persistent pyridyl radical anion, which subsequently is incorporated in radical–radical coupling reactions with other transient free radicals in the reaction system. A schematic of photoinduced radical–radical coupling of 4-cyanopyridines with alkenes and other radical sources is illustrated in Fig. 2.

1,2-Difunctionalizations of the electrophilic C=C bond of alkenes (especially styrenes) have been widely developed under visible light conditions.^17–19^ A variety of free radical precursors can generate free radicals *via* the SET strategy,^20^ EDA strategy,^21^ XAT strategy,^22^ MHAT strategy,^23^ and HAT strategy,^24^ which then react with alkenes and 4-cyanopyridine. In such photo-induced reactions, transition metal catalysts or non-metal catalysts have been employed as photocatalysts.

As a result, efficient and selective assembly of complex molecular architectures, including a pyridine core, from readily accessible feedstock has drawn the intensive attention of chemists. Given the ubiquitous value of the pyridine motif, especially in bioactive molecules, this review highlights photo-induced radical–radical coupling of alkenes with 4-cyanopyridines as a radical pyridylation reagent and other radical precursors.

## 2. Visible light-mediated reactions involving 4-cyanopyridines

### 2.1. C–C and C–C bonds formation

#### 2.1.1. Transition metal catalysis

**Scheme 1:**
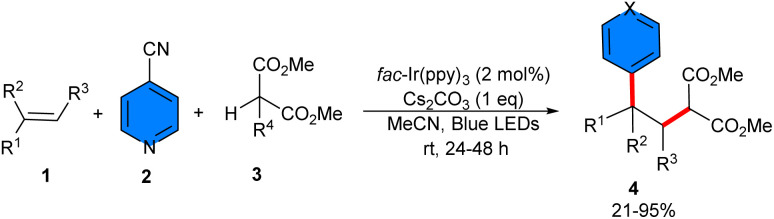
Ir-catalyzed reaction of alkenes, 4-cyanopyridines, and C(sp^3^)−H feedstocks.

**Scheme 2:**
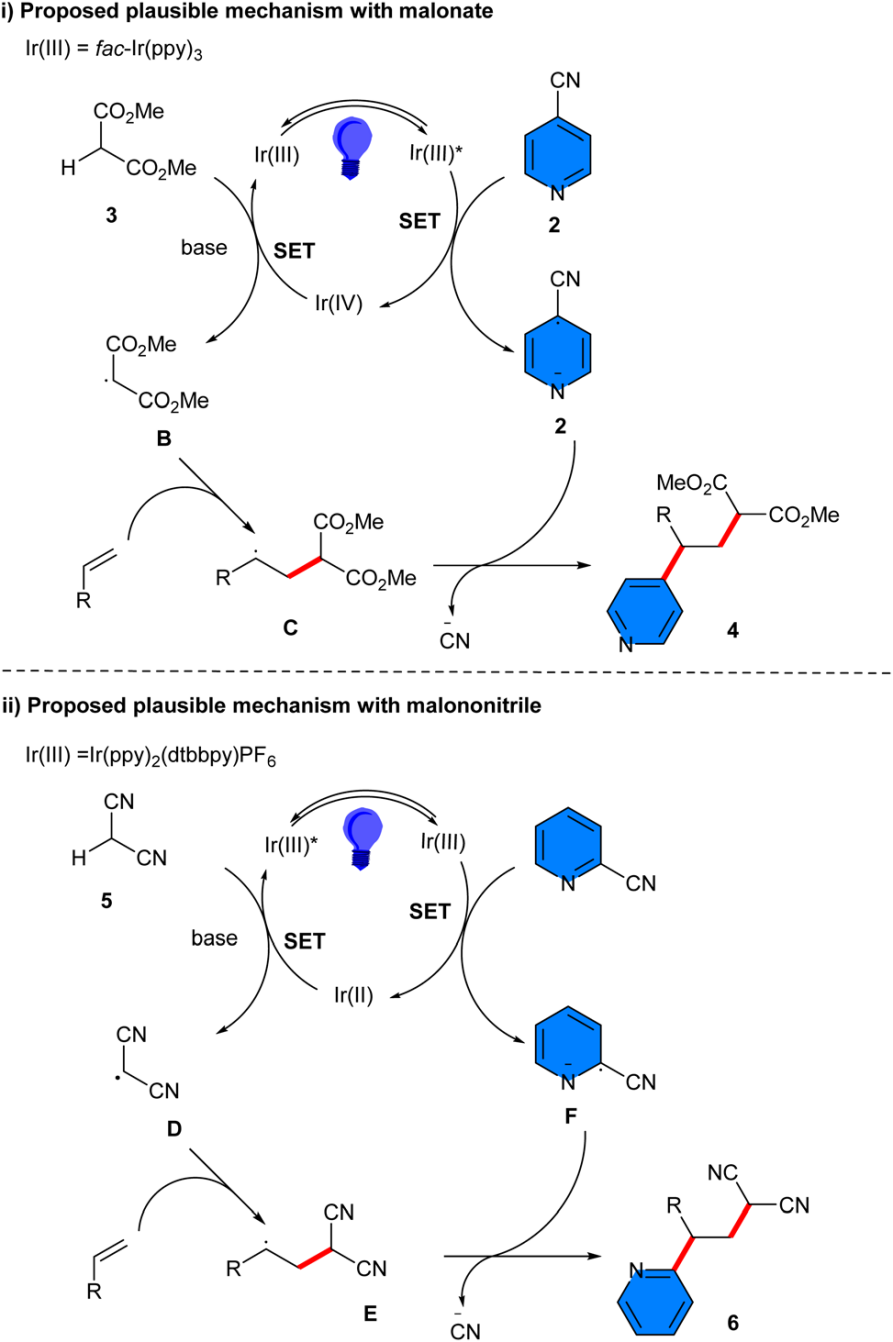
Plausible mechanism for Ir-catalyzed reaction of alkenes, 4-cyanopyridines, and C(sp^3^)−H feedstocks.

An iridium photocatalyst was used for carboarylation of unactivated alkenes 1 using 4-cyanopyridines 2 and a variety of protic C(sp^3^)−H feedstocks 3 (Scheme 1).^25^ The substrate scope with respect to the C(sp^3^)−H feedstocks were dialkyl malonates, and cyclic 1,3-diones. However, malononitrile could lead to the corresponding product in 56% yield using Ir(ppy)_2_(dtbbpy)PF_6_. To gain insight into the mechanism, a radical scavenger experiment using 2,2,6,6-tetramethylpiperidine-*N*-oxyl (TEMPO) was performed, resulting in complete inhibition of the reaction. Thus, a radical route was proposed to be involved, which started with the oxidation of *fac*-Ir(ppy)_3_* by 4-cyanopyridine to obtain the oxidized photocatalyst Ir(iv) and the radical anion A. Then, Ir(iv) oxidized the malonate anion, resulting in the malonate radical B, and *fac*-Ir(ppy)_3_. The electrophilic radical B was trapped by the alkene to generate radical C, which coupled with radical anion A to deliver the product 4 (Scheme 2i). For malononitrile 5 as a C(sp^3^)−H feedstock, the excited state photocatalyst was reduced by the malononitrile anion, producing the radical D and Ir(ii), which added to the alkene to yield intermediate E. On the other hand, Ir(ii) was oxidized by 2-cyanopyridine to form the radical anion F and regenerate the photocatalyst. Finally, radical F underwent radical–radical coupling with radical E to deliver the product 6 (Scheme 2ii). Furthermore, the late-stage functionalization of some complex molecules, including alkenes derived from clofibrate, ibuprofen, gemfibrozil and β-pinene, as well as some antiallergies such as pheniramine, chlorpheniramine, and brompheniramine also were performed in this work.

**Scheme 3:**
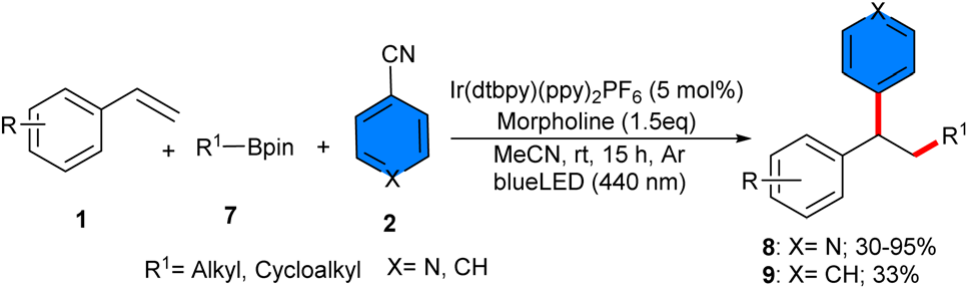
Ir-catalyzed reaction of styrenes, 4-cyanopyridines, and alkylboronic pinacol esters.

**Scheme 4:**
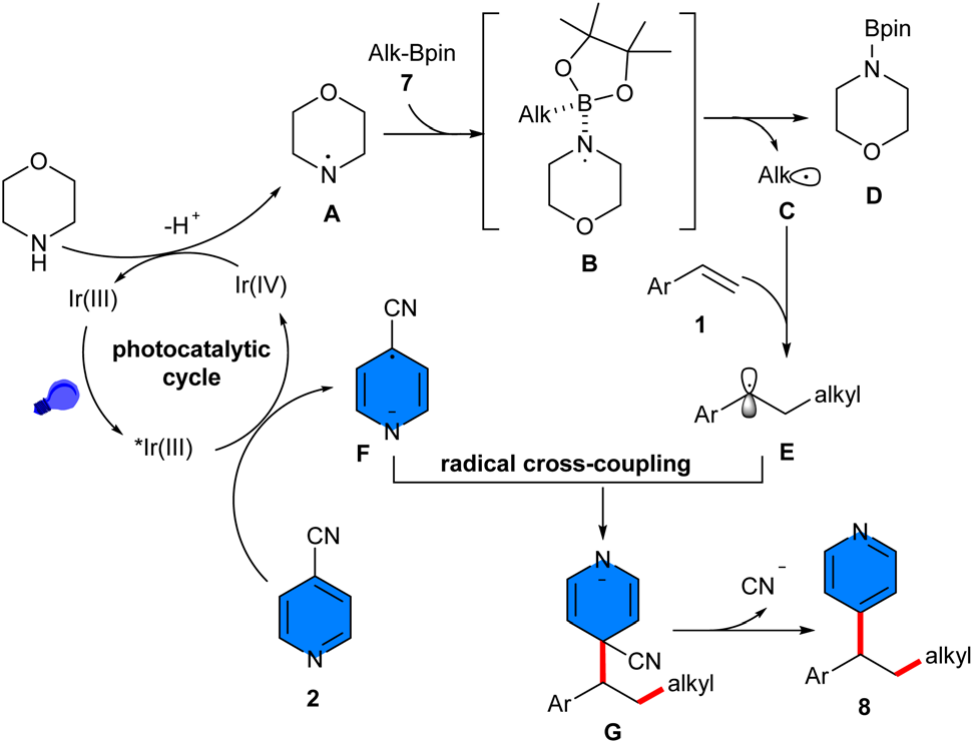
Possible mechanism for Ir-catalyzed reaction of styrenes, 4-cyanopyridines, and alkylboronic pinacol esters.

**Scheme 5:**
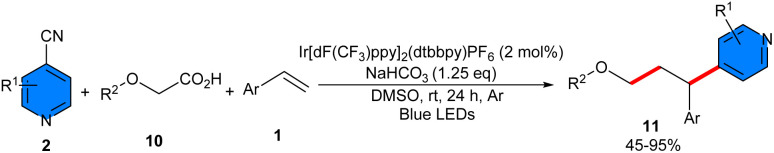
Ir-catalyzed reaction of styrenes, 4-cyanopyridines, and aryloxyacetic acid.

**Scheme 6:**
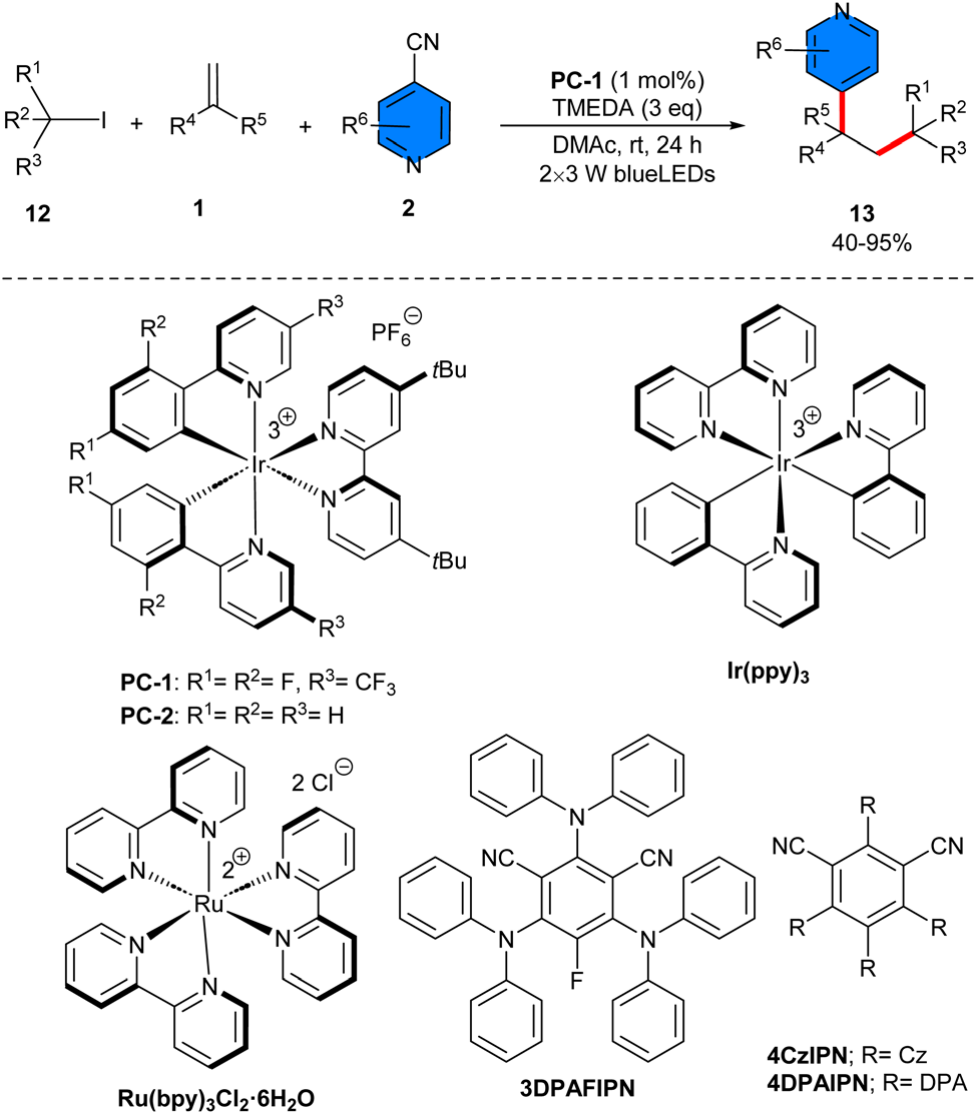
Ir-catalyzed reaction of alkenes, 4-cyanopyridines, and alkyl iodides.

Deoxygenative radical three-component reaction of styrenes 1, 4-cyanopyridines 2, and alkylboronic pinacol esters (APEs) 7 was carried out in the presence of an iridium catalyst under photo-redox conditions (40 W kessil blue LED) (Scheme 3).^26^ Investigation of iridium photocatalysts showed that Ir(dtbpy)(ppy)_2_PF_6_ gave the desired product in 67%, Ir(dFCF_3_ppy)_2_(bpy)PF_6_ yielded 59%, and *fac*-Ir(ppy)_3_ did not result in any product. 4CzIPN as an organocatalyst was also workable, affording the product in 49% yield. By the achievement of the optimal catalyst, a series of 1,1-diarylalkanes were well synthesized in moderate to excellent yields. In addition to the iridium photocatalyst, morpholine was found to be vital for this reaction. As an amino radical transfer (ART) reagent, morpholine was oxidized by Ir(iv) through the iridium cycle. The ART process between aminyl radical A and alkyl-Bpin 7 rendered alkyl radical C and amine-Bpin D. Then, C was trapped by styrene 1 to generate the benzyl radical E, which coupled with radical F to produce intermediate G, followed by decyanation to furnish the product 8 (Scheme 4). The scalability of this method was studied by expanding the scope of styrenes bearing electron-donating and electron-withdrawing groups, as well as primary, secondary and tertiary alkylboronic pinacol esters, which all were well tolerated in this radical transformation. Good results were also achieved for the substrate tolerance of 4-cyanopyridines with Cl and Me substituents at the *ortho*- and *meta*-positions, affording the corresponding products in 60–82% yields. Electronic and steric effects had negligible effects on this reaction. At the same time, Ir[dF(CF_3_)ppy]_2_(dtbbpy)PF_6_ was used for another three-component reaction involving styrenes 1, aryloxyacetic acid 10, and 4-cyanopyridines 2. A new series of 4-alkylpyridines containing aryl and aryloxy substituents on the phenyl ring was obtained in moderate to high yields (37–96%) under mild conditions using 3 W blue LEDs (Scheme 5).^27^ In this method [Ir(dtbbpy)(ppy)_2_]PF_6_ and [Ir(dF(Me)ppy)_2_(dtbbpy)]PF_6_ also gave the desired product in 64% and 32% yields. Evaluation of 4-cyanopyridines revealed that no electronic or steric effects affect their reactivities. However, it was different for aryloxyacetic acids, as electron-rich acids had higher product yields than electron-poor acids, implying that a higher electron donor ability of the carboxylate improves the reaction because the excited photocatalyst is reductively quenched by the carboxylate. The gram-scale synthesis of the product resulted in 1.327 gr, 72% yield. Experimental study of the radical intermediates and computation of the energy profile suggested that the mechanism involved the excited photocatalyst-oxidized aryloxyacetate anion, followed by decarboxylation to form aryloxymethyl radical, which added to styrene to produce another alkyl radical. Meanwhile, the photocatalyst reduced 4-cyanopyridine to the anion radical. Finally, radical–radical coupling gave the product and released a cyanide anion. The same iridium photocatalyst successfully catalyzed 1,2-alkylpyridylation of alkenes 1 with 4-cyanopyridines 2 and alkyl iodines 12 (Scheme 6).^28^ The reaction was carried out in the presence of only 1 mol% Ir[dF(CF_3_)ppy]_2_(dtbbpy)PF_6_ as a photocatalyst, *N*,*N*,*N*,*N*-tetramethylethylenediamine (TMEDA) as a terminal reductant and 2 × 3 W blue LEDs at room temperature, all of which were critical parameters for this transformation. Other photocatalysts such as Ir(ppy)_3_, Ru(bpy)_3_Cl_2_·6H_2_O, 4CzIPN, 3DPAFIPN, or 4DPAIPN resulted in inferior yields of the product (29–70%). 4-Cyano-substituted pyridines bearing electronically diverse substituents at the C2 and C3 positions reacted smoothly with mono and disubstituted styrenes, and primary, secondary, and tertiary alkyl iodides.

**Scheme 7:**
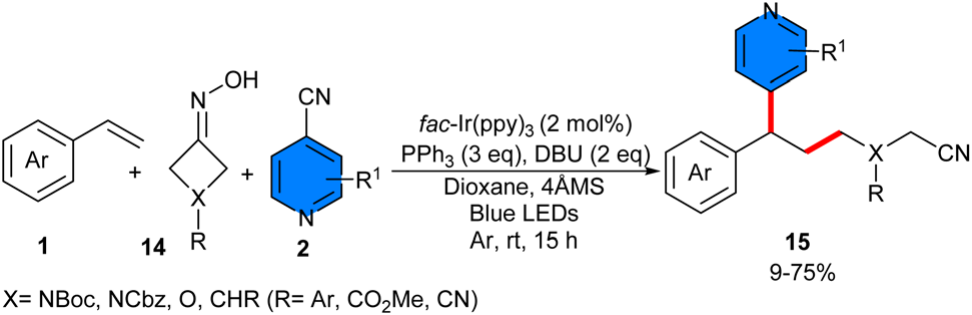
Ir-catalyzed reaction of styrenes, 4-cyanopyridines, and cyclic oximes.

**Scheme 8:**
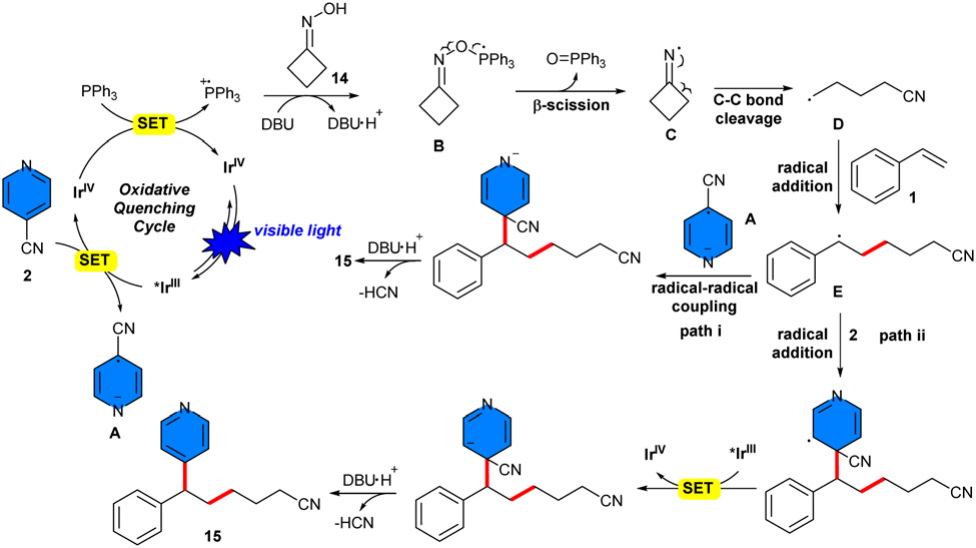
Catalytic cycle for Ir-catalyzed reaction of styrenes, 4-cyanopyridines, and cyclic oximes.

In 2023, Liu *et al.* reported regioselective three-component 1,2-cyanoalkylpyridylation of styrenes 1 utilizing 4-cyanopyridine 2 and accessible nonredox-active cyclic oximes 14*via fac*-Ir(ppy)_3_ as a visible-light photoredox catalyst in the presence of PPh_3_ as a ligand and DBU as a base, resulting in the formation of various β-cyanoalkylated alkylpyridines (Scheme 7).^29^ In this research, Liu *et al.* evaluated several photocatalysts such as *fac*-Ir(ppy)_3_, Ir(p-F-ppy)_3_, Ir(dFppy)_3_, Ru(bpy)_3_(PF_6_)_2,_ where *fac*-Ir(ppy)_3_ was the best photocatalyst. According to the previously mentioned experiments, in the more favored path I, firstly, the photoexcited *fac-Ir(ppy)_3_ was oxidatively quenched by 4-cyanopyridine 2, resulting in the formation of *fac*-Ir^IV^(ppy)_3_ species and a persistent radical anion A. PPh_3_ was oxidized by the *fac*-Ir^IV^(ppy)_3_ species to form its corresponding radical cation, concomitant with the regeneration of the ground-state *fac*-Ir(ppy)_3_. With DBU present, the phosphoryl radical species B was formed through a polar-radical crossing between the phosphoryl radical cation and cyclobutanone oxime 14. Subsequently, β-selective N–O bond cleavaged to form triphenylphosphineoxide, followed by strain-relieved homolytic C–C bond cleavage, occurring sequentially to generate the translocated cyanoalkyl radical D. Later, radical D could be selectively attached to styrene 1 to form benzylic radical E. Ultimately, product 15 was formed through an intermolecular radical–radical coupling between radical E and persistent radical anion A, accompanied by the release of the cyano anion. Another potential pathway, II, involved the radical addition of 4-cyanopyridine 2 to benzyl radical E, followed by SET with the photoexcited photocatalyst and elimination of cyanide anion to yield the product 15 (Scheme 8). Typically, the reaction was not significantly affected by the substitution patterns and electronic characteristics of styrenes. 3-Substituted symmetrical cyclic oximes, which have a functional group like cyano, ester, *para*-substituted phenyl, or protected amino, were well tolerated. Notably, this method enabled late-stage functionalization of biologically active molecules. Furthermore, a scaled-up model reaction was conducted to obtain the target alkylpyridine (172.7 mg, 69%).

**Scheme 9:**
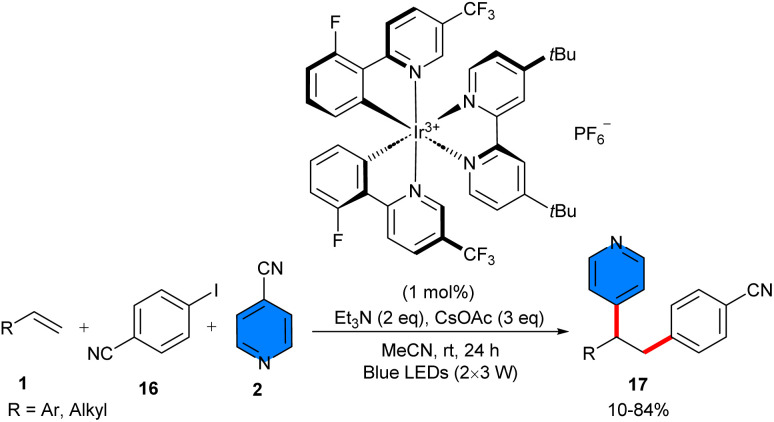
Ir-catalyzed reaction of alkenes, 4-cyanopyridines and aryl halides.

**Scheme 10:**
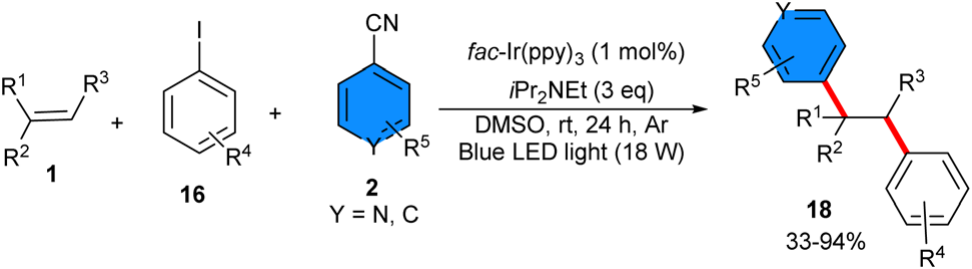
Ir-catalyzed reaction of alkenes, 4-cyanopyridines and aryl halides.

Photoredox three-component 1,2-heterodiarylation of alkenes 1 with aryl iodides 16 and 4-cyanopyridines 2 was accomplished in the same year (Scheme 9).^30^ The Ir catalyst (1 mol%) was used as an organophotocatalyst to make a series of polyarylalkanes in a highly regioselective fashion. The gram-scale synthesis (11.3 gr, 76%), and 1,2-diarylation of several biologically active alkenes demonstrated the potential application of this methodology. Primary investigations offered the involvement of C(sp^3^)-radicals, and the presence of tertiary alkylamine as the terminal reductant effectively prevented the further oxidation of these C-radicals to the carbocations, allowing cascade radical process. In addition, halogen-atom transfer (XAT) between aryl iodides and nucleophilic α-aminoalkyl radicals produced transient aryl radicals, while a SET of aryl cyanides and reduced 4CzlPN gave persistent aryl radical anions; both were important to achieve the radical relay sequence in a highly regioselective manner. Another 1,2-diarylation of alkenes 1 with aryl halides 16 and 4-cyanoaromatics 2 could also be carried out under iridium photocatalytic system (Scheme 10).^22^ A broad range of terminal and internal alkenes reacted smoothly with electron-donating and electron-withdrawing aryl iodides. It should be noted that their steric hindrance of aryl iodides affected their reactivity, but their electron nature had no effect. Besides, bromobenzene and chlorobenzene were also well tolerated in this coupling reaction. Both electron-deficient 4-cyanopyridine and benzonitrile were compatible under these conditions. *fac*-Ir(ppy)_3_ acted as a photocatalyst, and *i*Pr_2_NEt served as an electron transfer agent. Aryl halides selectively underwent halogen atom transfer (XAT) to generate the aryl radicals, thus, two new C(sp^2^)-C(sp^3^) bonds between the cabron atoms were formed in a radical addition, radical–radical coupling, and decyanation, assembling a wide array of functionalized polyarylalkanes with high regio- and chemoselectivity.

**Scheme 11:**
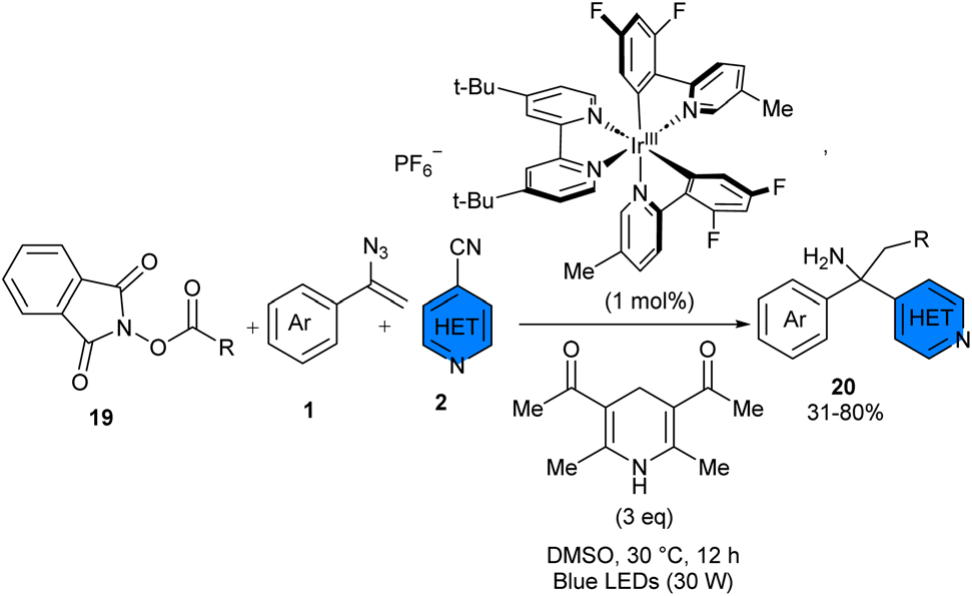
Ir-catalyzed reaction of alkenes, 4-cyanopyridines, and NHPI.

In 2024, Davies and co-workers found that combining Hantzsch ester (HE-1) and iridium complex (PC-1) can efficiently catalyze alkylarylation and dialkylation of vinyl azides 1 by redox-active *N*-hydroxyphthalimide (NHPI) esters 19 and 4-cyanoarene 2 or aryl aldehyde, respectively (Scheme 11).^31^ The presence of HE-1 and visible light was crucial for the reaction, while the absence of PC-1 resulted in a decreased product yield (72%). The reaction proceeded under blue LEDs and mild conditions, providing access to structurally diverse α,α,α-trisubstituted primary amines. Vinyl azide as a precursor of primary amine was enabled by the dual role of the Hantzsch ester to form an electron donor–acceptor complex and serve as a sacrificial reductant. The synthetic utility of the method was demonstrated by the gram-scale synthesis of the product (1.16 gr, 66% yield), and the preparation of 2,2-disubstituted tetrahydroquinolines. Mechanistic investigations suggested that two parallel reductive photocatalytic cycles were possible, allowing the denitrogenative alkylarylation or dialkylation of vinyl azides, followed by coupling of α-amino radicals with aryl anion radicals or ketyl anion radicals.

**Scheme 12:**
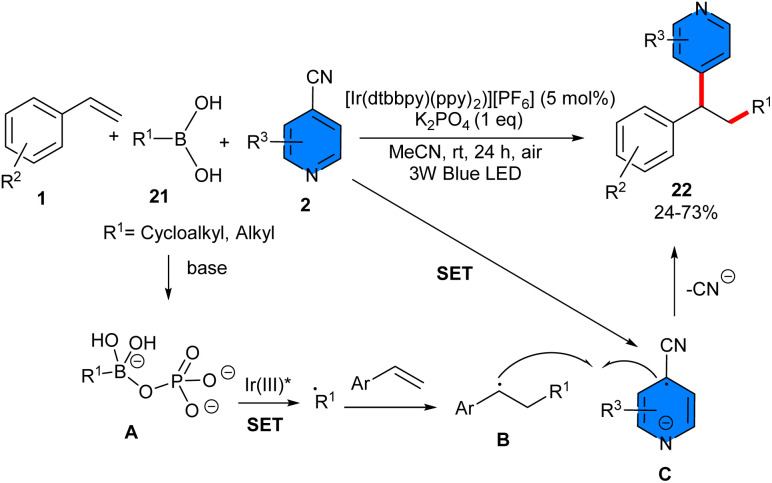
Ir-catalyzed reaction of alkenes, 4-cyanopyridines, and alkylboronic acids.

In 2025, alkylpyridylation of alkenes 1 with 4-cyanopyridines 2 and alkylboronic acids 21 was reported by Shen *et al.* (Scheme 12).^32^ Investigating of various catalysts indicated that Ir[dF(CF_3_)ppy]_2_(dtbbpy)PF_6_ [Ir(dtbbpy)(ppy)_2_][PF_6_], and Ir(ppy)_3_ afforded the desired product in 20–30% yields in CH_2_Cl_2_ as a solvent. In contrast, other catalysts such as Ru(bpy)_3_(PF_6_)_2_, 4CzIPN, and Eosin Y were not workable. As a result, the optimal condition was achieved using [Ir(dtbbpy)(ppy)_2_][PF_6_] (5 mol%), and K_3_PO_4_ (1 eq) in acetone at room temperature under 3 W blue LEDs. Generally, electron-rich styrenes afforded the corresponding products in higher yields than electron-poor styrenes. This radical transformation was remarkably affected by the steric effect in respect to styrenes and 4-cyanopyridines. Where *ortho*-substituted styrene showed lower reactivity than unsubstituted or *para*-substituted styrenes, and *meta*-substituted 4-cyanopyridines afforded higher product yields than ortho-substituted 4-cyanopyridines, although no significant electronic effects were observed in the case of 4-cyanopyridines. For boronic acids bearing primary and secondary alkyls with different lengths and structures all were compatible. Radical scavengers: TEMPO and BHT, as well as the energy profile of the reaction, confirmed a radical pathway involving the SET process between complex A and Ir(iii)* to form the alkyl radical, followed by sequential radical addition to the alkene, and radical addition to the radical anion C. The synthetic application of this procedure was illustrated by the gram-scale preparation of two derivatives of the product (0.912 gr, 68%, and 0.929, 69%).

**Scheme 13:**
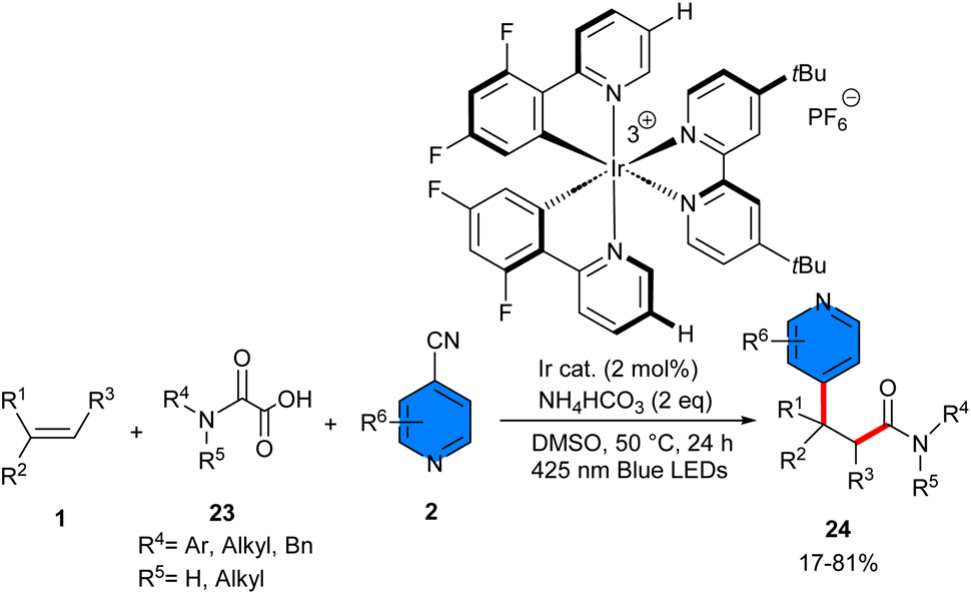
Ir-catalyzed reaction of alkenes, 4-cyanopyridines, and oxamic acids.

An iridium photocatalyst was utilized for the pyridyl-carbamoylation of alkenes 1 using 4-cyanopyridines 2 and oxamic acids 23 (Scheme 13).^33^ A series of β-pyridylamides were synthesized in up to high yields with high chemoselectivities. Alkenes, oxiamic acids, and 4-cyanopyridines could all participate efficiently in this transformation, and the electronic properties and substitution patterns exhibited limited impact. For instance, different substituents (OMe, Cl, Br, Ph, CF_3_) on the C2 position of 4-cyanopyridines, 3-Me-substituted 4-cyanopyridine, and 4-cyano-isoquinoline were all viable candidates for this synthetic method. In the case of styrenes, electron-donating, electron-neutral, and electron-withdrawing substituents regardless of *para*-, *meta*- or *ortho*-positions invariably engaged smoothly to deliver moderate to good product yields. Apart from styrenes, 1,2-disubstituted internal alkenes were also compatible with this iridium catalytic system. Furthermore, the examination of alkyl alkenes could give more detail about the chemoselectivity of this reaction. For example, 2-ethyl-1-butene yielded the corresponding product in a 14% yield, while 1-hexene did not undergo the transformation. According to Stern–Volmer quenching experiment, the excited-state iridium photocatalyst underwent an SET process with oxamic acid under basic conditions, indicating the presence of the carbamoyl radical intermediates. Consequently, it was found that the stability of the radical intermediate formed after the addition of the carbamoyl radical to the alkene can affect the direction of the transformation. As highly stable benzylic radicals have a greater tendency to couple with pyridyl radical anions compared to less stable alkyl radicals.

#### 2.1.2. Organocatalysis

**Scheme 14:**
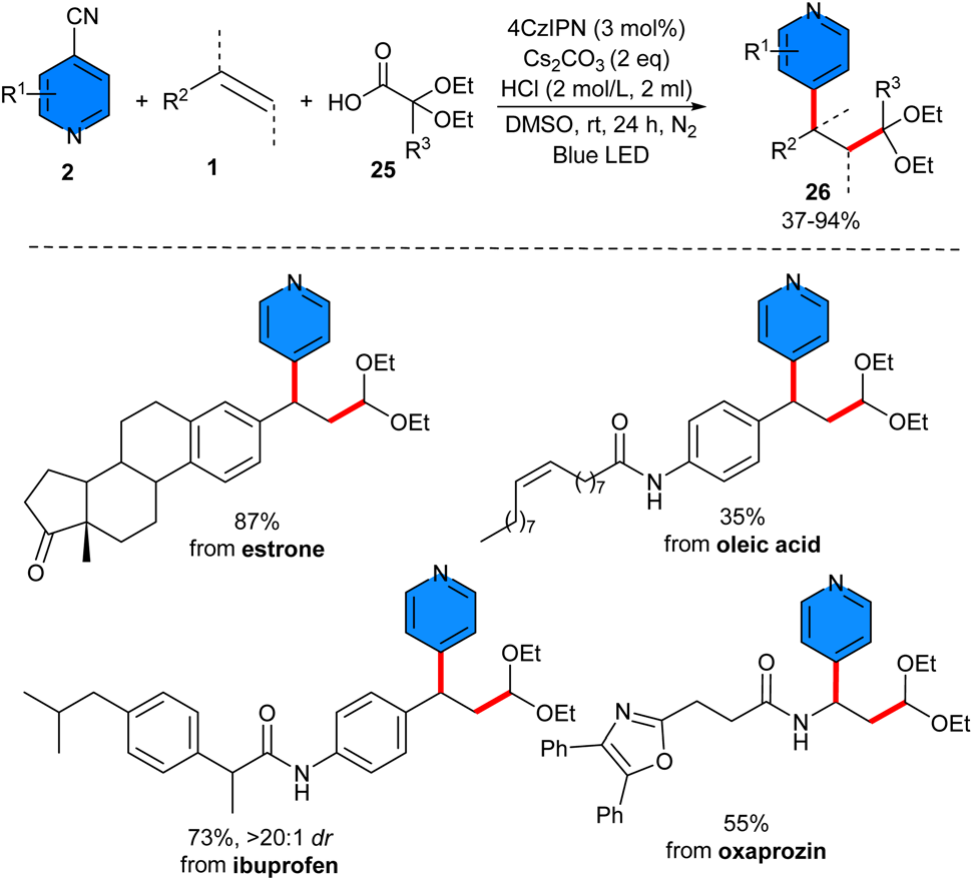
Photo-induced 4CzIPN-catalyzed reaction of alkenes, 4-cyanopyridines, and sodium sulfinates.

In 2022, Zhang and co-workers developed an organocatalysis system for the pyridylation of alkenes 1 with 4-cyanopyridines 2 and diethoxyacetic acids 25 (Scheme 14).^34^ Among various organocatalysts, 4CzIPN was chosen as the optimal catalyst under this visible light condition. The synthetic utility of this method was demonstrated by late-stage modification of bioactive molecules, including estrone, oxaprozin, ibuprofen, and oleic acid, as well as the antitumor activity of the products. Further transformations of the aldehyde unit into valuable functional groups, such as carboxylic acid, alcohol, and alkene, were also reported in this work.

**Scheme 15:**
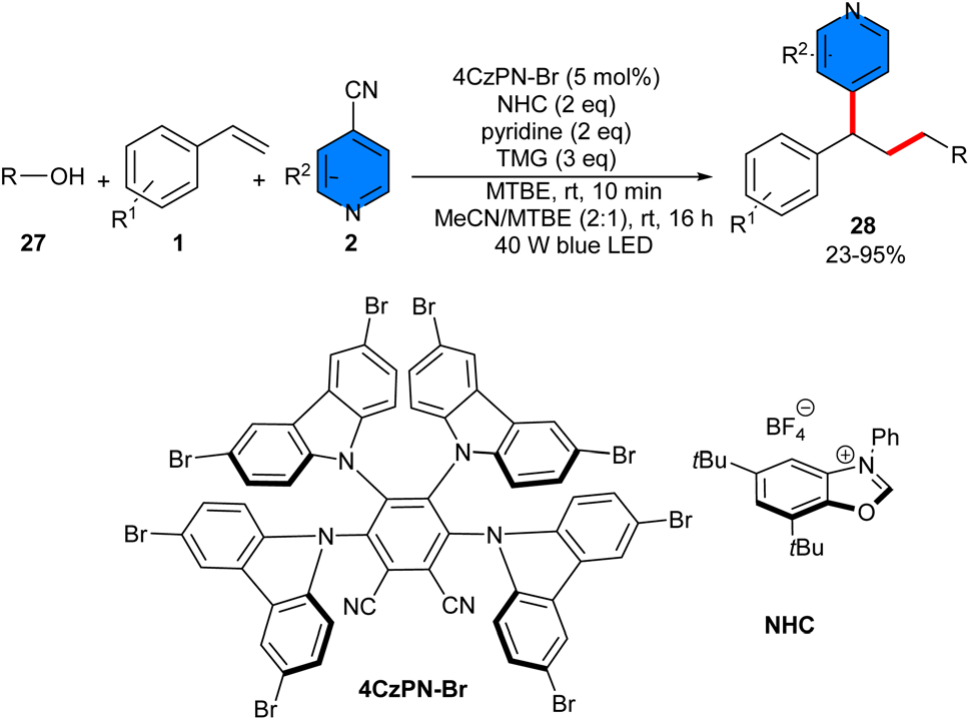
NHC-catalyzed reaction of styrenes, 4-cyanopyridines, and alcohols.

The use of an NHC catalyst catalyzed efficiently the deoxygenative reaction of styrenes 1, 4-cyanopyridines 2, and alcohols 27 (Scheme 15).^35^ Among various organocatalysts, including 4CzIPN, 4CzIPN-Br, Ir(ppy)_2_(dtbbpy)PF_6_, and NHC catalyst, it was found that NHC is the optimal catalyst to activate alcohols towards alkyl radicals to participate in a radical relay mechanism, involving the Giese addition of aryl alkenes by alkyl radicals, followed by the decyanative pyridination of the benzyl radicals. The presence of 40 W blue LEDs, the NHC photocatalyst and etramethylguanidine (TMG) as the base, were all necessary for the reaction to proceed. A wide variety of primary, secondary, and tertiary alcohols were well tolerated in this reaction. The reaction affected by the electronic effects in the case of alkenes, wherein electron-neutral and *para*-electron-donating groups afforded the corresponding products in good to high yields, while a notable electronic effect was observed for halogen groups; the F group represented higher efficiency than Cl or Br groups. 4-Cyanopyridines with substituents at the different positions were well transformed into the target products. Several control experiments, including radical trapping experiments using TEMPO and 1,1-diphenylethylene, and radical clock experiment suggested a radical pathway. In addition, the late-stage modification of complex molecules was applicable in this methodology.

**Scheme 16:**
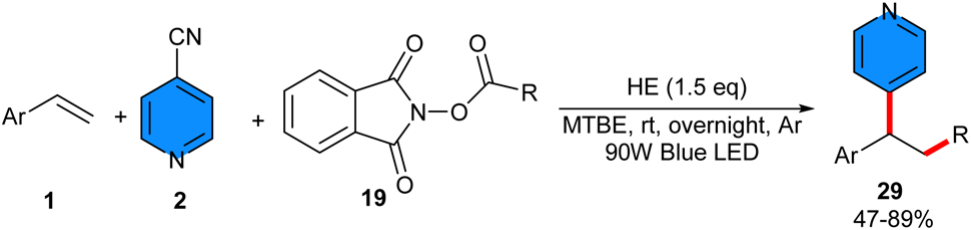
Metal-free reaction of alkenes, 4-cyanopyridines, and Hantzsch ester.

**Scheme 17:**
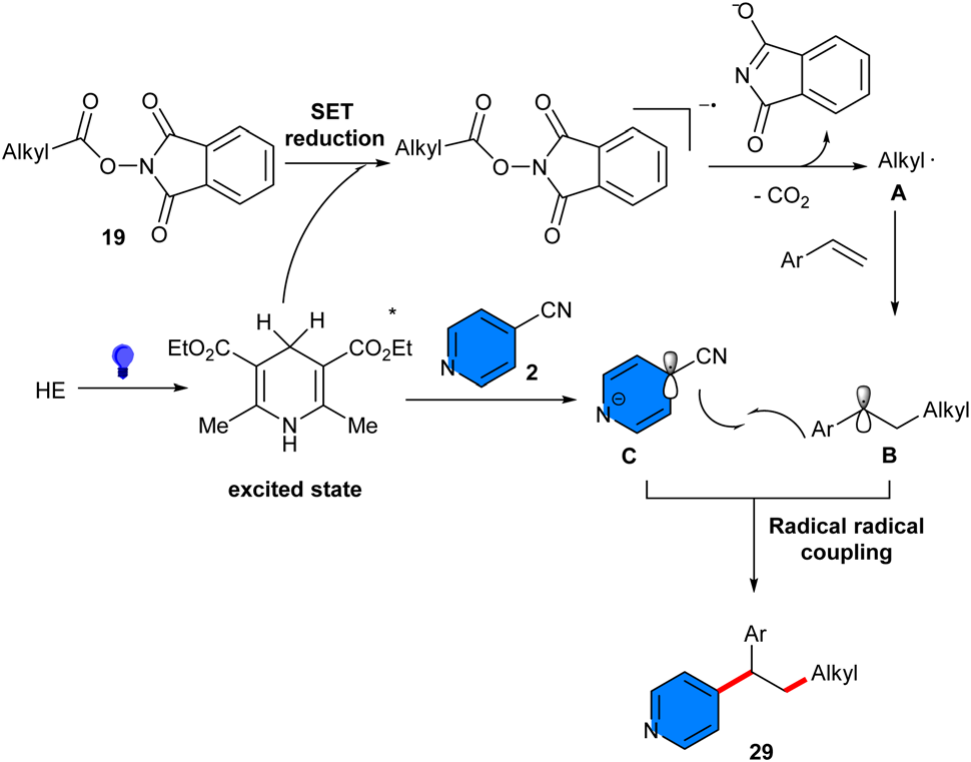
Plausible mechanism for metal-free reaction of alkenes, 4-cyanopyridines, and Hantzsch ester.

In 2021, a metal-free reductive radical coupling reaction involving 4-cyanopyridine 2, NHP ester 19, and styrene 1 was developed by Chen *et al.* (Scheme 16).^36^ In this regard, the authors employed visible-light irradiation to activate the Hantzsch ester as a radical initiator. To prove the radical mechanism, a radical scavenger, 2,2,6,6-tetramethyl-1-piperidinyloxy (TEMPO), was used, which totally inhibited the reaction process. As shown in Scheme 4, the reaction started with a single-electron reduction of photoexcited HE* with NHP ester access to alkyl radical A. Subsequently, A was trapped by styrene to generate benzylic radical B. Simultaneously, reduction of 4-cyanopyridine by the excited state of photoexcited HE* *via* a SET process generated radical anion C, which coupled with radical B to deliver the alkylpyridine product 6*via* the extrusion of a CN motif (Scheme 17). Furthermore, this reaction could be expanded into 2-cynaoquinoline and 1-cynaoisoquinoline as alternative substrates for 4-cyanopyridine, leading to 42% and 66% yields, respectively.

**Scheme 18:**
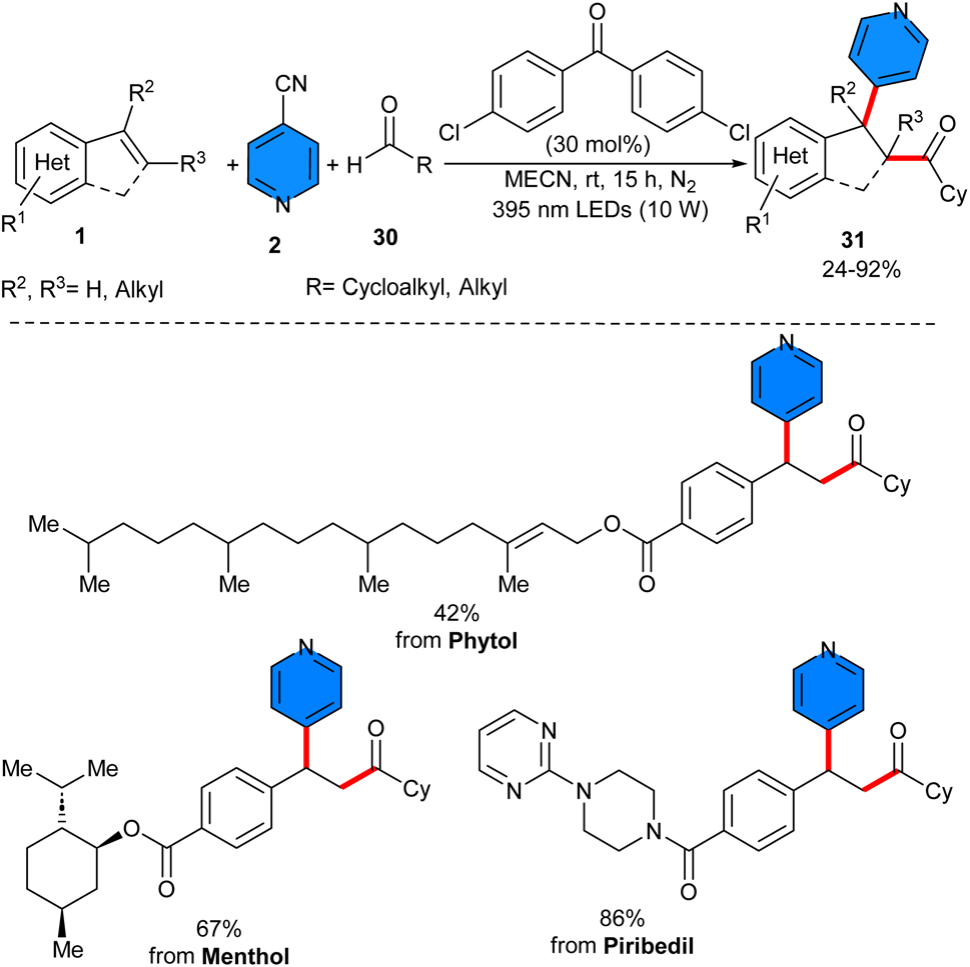
Aryl ketone-catalyzed reaction of vinylarenes, 4-cyanopyridines, and aldehydes.

Under metal-free photocatalytic conditions, a series of vinylarenes 1 became functionalized with 4-cyanopyridines 2 and alkyl aldehydes 30 (Scheme 18).^37^ The presence of diaryl ketone as a non-metal catalyst (4,4′-dichlorobenzophenone) and visible light (10 W blue LEDs) both were essential for the reaction to proceed. Various alkyl and cycloalkyl aldehydes, as well as aldehydes connected with hydroxy, amide, and alkene moieties were well converted to the target products. Trimethylacetaldehyde as an acyl precursor led to only a decarbonylated product, implying more stability of the tertiary alkyl radical. When a C–H precursor with both activated C(sp^3^)-H bonds and a formyl C(sp^2^)-H bond was used, the latter bond remains intact, confirming the inactivity of aromatic aldehydes by having reduced hydridic property of formyl C–H bonds. The tolerance of vinylarenes containing electron-withdrawing and electron-donating groups as well as 1,1-disubstituted olefin, internal olefins, vinylarene containing a terminal alkynyl group, and vinyl heterocycles all were suitable reactants. Only 4-cyanopyridine worked, which was a main limitation of this synthetic method. The resulting β-pyridinyl ketones products could be easily transformed into corresponding alcohols. The method was scalable, resulting in the formation of the product in moderate yield (1.25 gr, 49%). Moreover, the potential application of this method was demonstrated by the functionalization of natural products and pharmaceutical molecules. TEMPO and BHT completely inhibited the reaction, indicating the involvement of a radical pathway. Using vinylcyclopropane instead of vinylarene for a radical clock experiment gave a ring-opening product in 66% yield as a mixture of *Z*/*E* isomers, which confirmed the presence of acyl-centered radical intermediate, based on these results and a HAT mechanism. The acyl radical was produced *via* polarity-matched HAT between alkyl aldehydes and a triplet-state diradical from benzophenone.

**Scheme 19:**
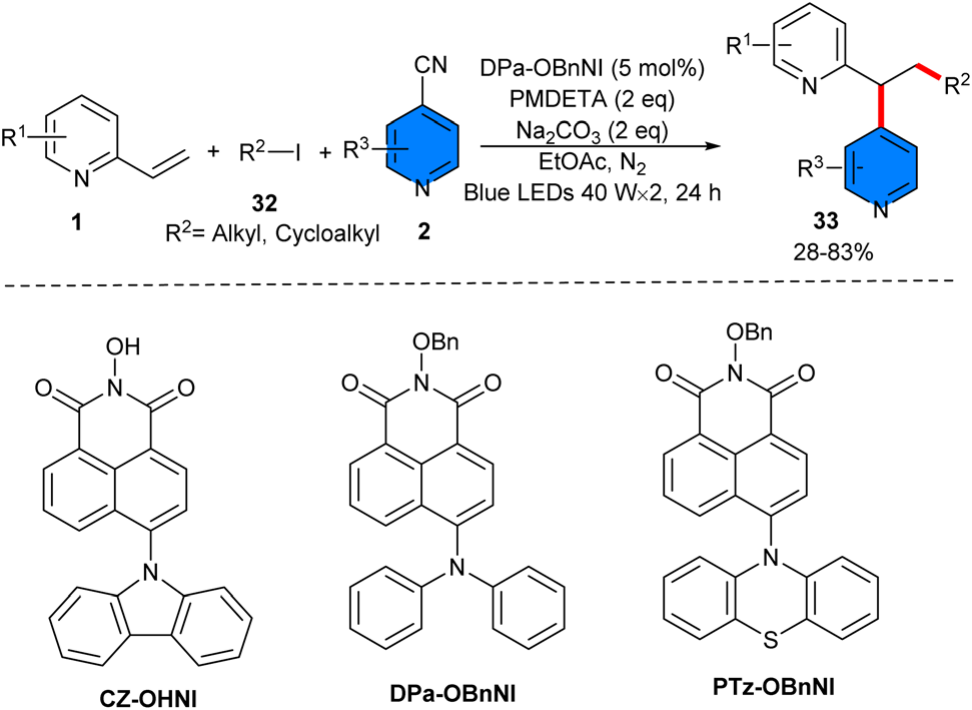
Organocatalytic reaction of alkenes, 4-cyanopyridines, and alkyl iodides.

Alkylarylation of alkenes and alkylation of acrylamides was reported by Xian and Ren in 2024 (Scheme 19).^38^ By examining several naphthalimide (NI)-based photocatalysts, the authors found that 5 mol% of DPa-OBnNI can efficiently catalyze alkylarylation of alkenes 1 with alkyl iodides 32 and 4-cyanopyridines 2 in the presence of 2 eq. of *N*,*N*,*N*′,*N*″,*N*″-pentamethyldiethylenetriamine (PMDETA) as a sacrificial reductant. This radical transformation required irradiation of 2 × 40 W blue LEDs and temperature of 37–40 °C to proceed. Under optimized conditions, 2-vinylpyridines showed good reactivity in the reaction with acyclic/cyclic alkyl iodides and 4-cyanopyridine. However, the scope of 4-cyanopyridines was limited, and none8 of the *ortho*-substituted 4-cyanopyridines were successful. Only methyl 2-cyanoisonicotinate showed inferior reactivity with a 31% yield of product. For alkyl radical addition/cyclization of acrylamides, 3 eq. of the reductant were required to drive the reaction *via* the XAT mechanism, which was later confirmed by radical trappers such as TEMPO and BHT.

**Scheme 20:**
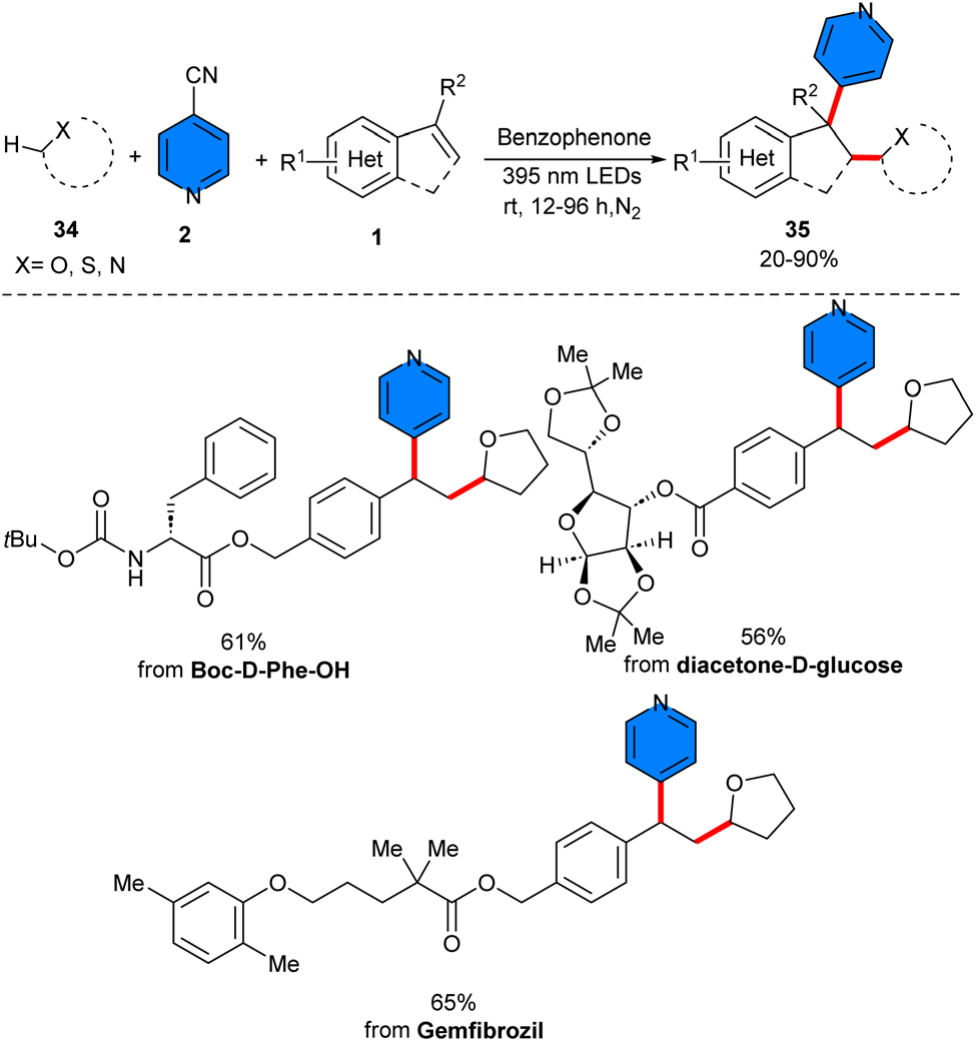
Benzophenone-catalyzed reaction of styrenes, 4-cyanopyridines, and cyclic ethers.

**Scheme 21:**
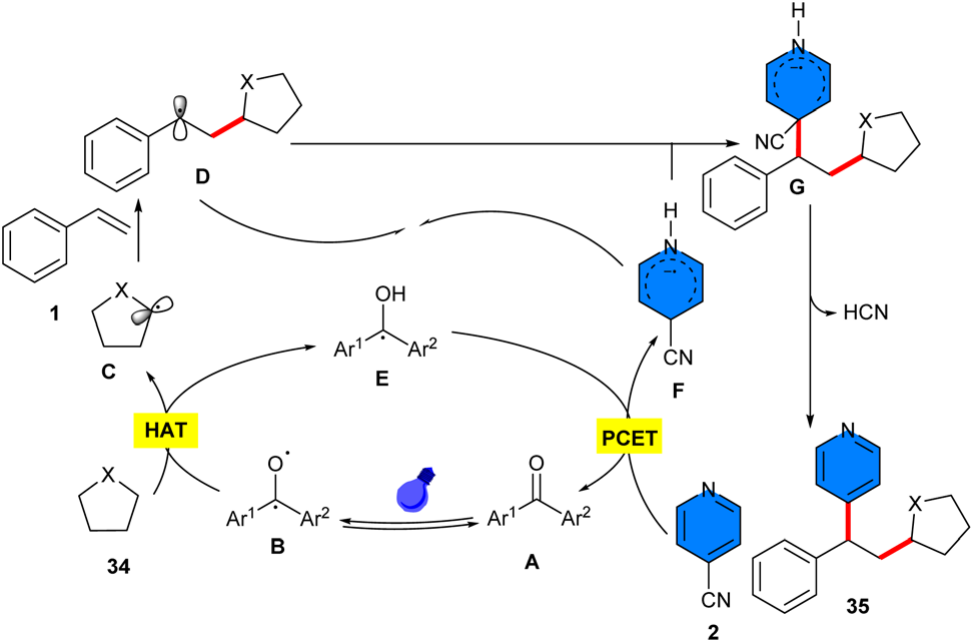
Plausible mechanism for benzophenone-catalyzed reaction of styrenes, 4-cyanopyridines, and cyclic ethers.

**Scheme 22:**
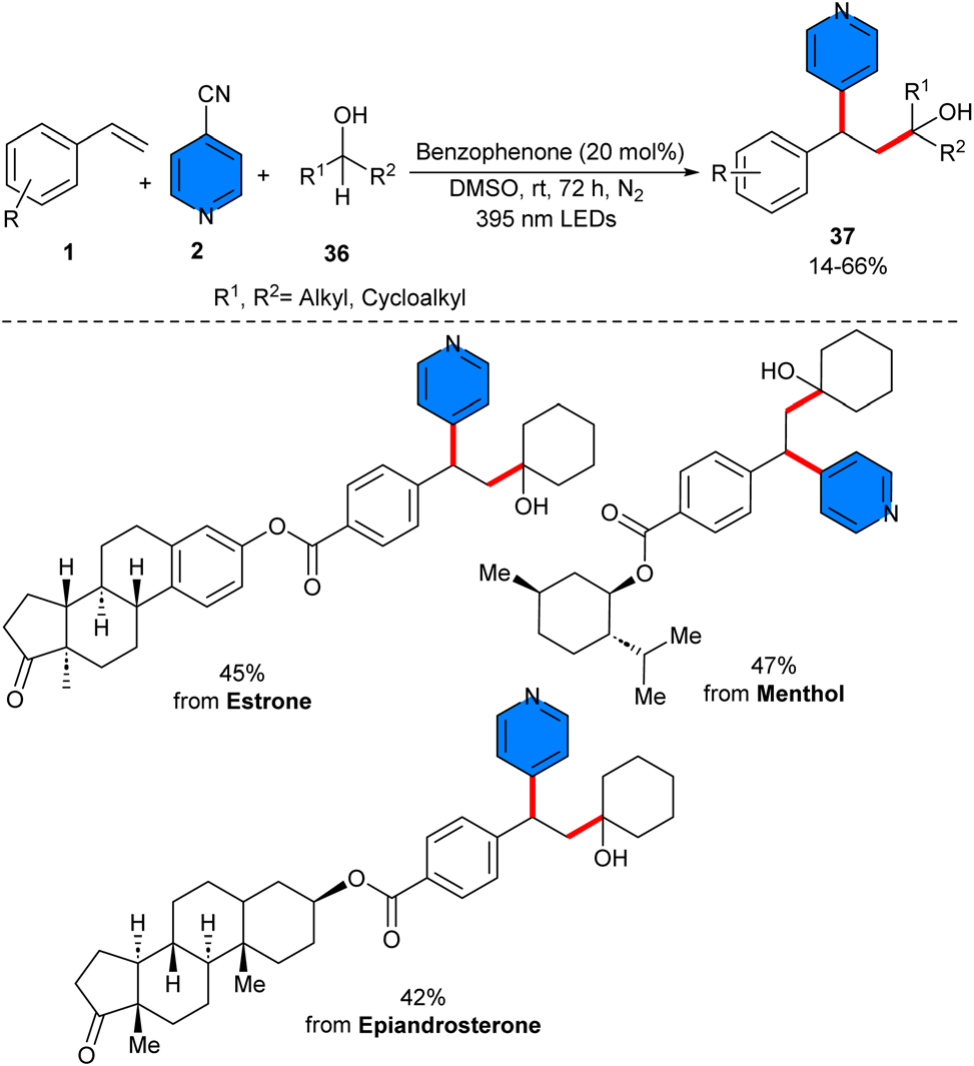
Benzophenone-catalyzed reaction of alkenes, 4-cyanopyridines, and tertiary alcohols.

Liu and co-workers suggested that a simple and cheap benzophenone molecule can catalyze three-component reaction of 4-cyanopyridine 2 with styrenes 1, and C–H precursors 34 (Scheme 20).^39^ In this protocol, benzophenone served as a hydrogen atom transfer (HAT) catalyst. Various C–H precursors, including THF, pyrrolidine, tetrahydrothiophene, aldehydes, cyclohexane, and isopropyl alcohol were well tolerated in this method. However, the substrate scope with respect to 4-cyanopyridine was not reported in this work. Considering the results of radical trapping and radical clock experiments, a plausible mechanism was proposed for this reaction involving the excitation of benzophenone to the oxygen radical, followed by a kinetically selective HAT reaction with saturated heterocycle 34 at the electron-rich site, leading to the radical C and ketyl radical E. Afterwards, C selectively added to styrene, generating the radical D. On the other hand, E underwent SET with 4-cyanopyridine 2, regenerating A and forming radical anion F. Eventually, radical–radical coupling of D and F provided the product 35*via* the extrusion of a cyanide anion (Scheme 21). Moreover, a variety of bioactive natural compounds and pharmaceutical molecules could be undergoing late-stage functionalization to display the potential pharmaceutical utility of this work. At last, the gram-scale reaction afforded 2.02 gr, 80% yield of product. Another use of benzophenone as a non-metal catalyst was found in the three-component reaction of alkenes 1, 4-cyanopyridines 2, and tertiary alcohols 36 (Scheme 22).^40^ The method involved α-functionalization of alcohols access to *γ*-pyridyl alcohol derivatives. Both electron-attracting groups and electron-donating substituents on the phenyl ring of styrenes, exhibited satisfactory performance in this transformation. Although the scope of the substrates in respect to 4-cyanopyridine was limited and the effect of functional groups on the pyridine ring was not reported. Like the proposed mechanism by Liu in the previous work, the photoexcited benzophenone abstracted the α-C–H, generating α-hydroxy radical. Next, α-hydroxy radical added to styrene, affording benzyl radical, which underwent the SET process with 4-cyanopyridine, providing cyano-pyridine radical, with the regeneration of benzophenone. Final radical–radical coupling of cyano-pyridine radical and benzyl radical produced the product upon liberation of HCN. Moreover, the late-stage functionalization of pharmaceutical molecules was also performed in this work.

#### 2.1.3. Base catalysis

**Scheme 23:**
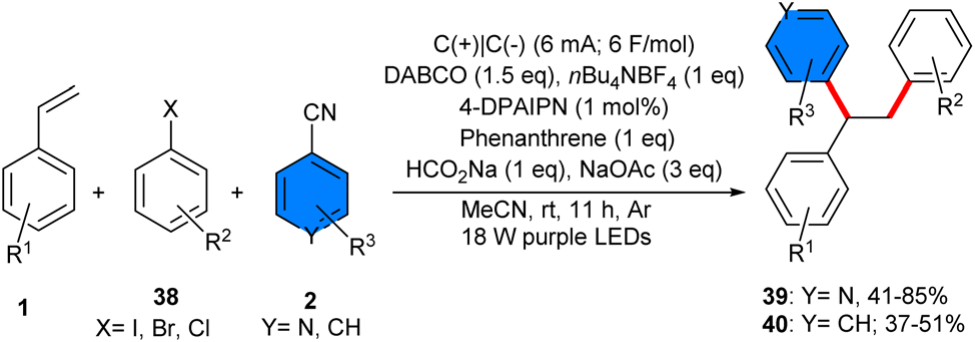
Electrophoto-mediated metal-free reaction of alkenes, 4-cyanopyridines, and aryl halides.

**Scheme 24:**
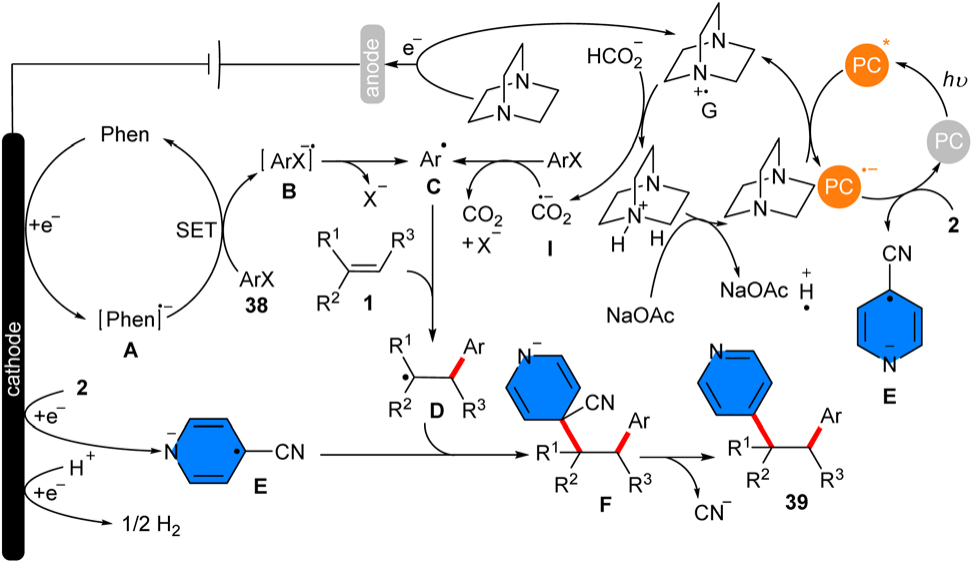
Possible mechanism for electrophoto-mediated metal-free reaction of alkenes, 4-cyanopyridines, and aryl halides.

In 2023, an electrophotocatalytic strategy for the 1,2-diarylation of alkenes 1 with aryl halides 38 and 4-cyanoaromatics 2 was introduced by Hu and co-workers (Scheme 23).^20^ Their radical–radical coupling reaction featured high regioselectivity, broad substrate scope, and high yields of products (up to 85%). No radical sacrificial anode was used in undivided cells, proposing a green and simple method for the synthesis of a diverse range of polyarylated alkenes (67 derivatives). Stern–Volmer fluorescence quenching experiments of 4-DPAIPN with DABCO indicated that the mechanism proceeds *via* a canonical photoredox cycle involving reductive quenching with DABCO. The Hammett plot investigation suggested the generation of a negative charge on the iodobenzene scaffold. Cyclic voltammetry (CV) study indicated that isonicotinonitrile was reduced more readily than phenanthrene. Phenanthrene acted as a mediator to promote the reaction by generating a reaction layer detached from the electrode, allowing the reduction of aryl halides. The DFT calculation confirmed the radical path of the reaction by showing favored transition states. Consequently, a radical reductive mechanism was proposed for this electrophoto-induced C–C coupling reaction. Firstly, reduction of phenanthrene occurred in cathode generating a phenanthrene radical anion A that underwent reductive ET with aryl iodide 38 producing aryl radical anion B. Alternatively, reductive quenching of the excited state PC* by DABCO gave the reduced PC radical anion and the DABCO radical cation G. Through a H-transfer between HCO_2_Na and G, the CO_2_ radical anion I and the DABCO cation H were generated. Then, the aryl halide was reduced by I, affording the aryl radical anion B, which dehalogenated to form the aryl radical C. The reaction of C with alkene 1 rendered the alkyl radical intermediate D, followed by radical–radical coupling with the cyanoaromatic radical anion E to obtain the alkyl cyanoaromatic anion intermediate F, which finally furnished the product 39 after the decyanation (Scheme 24).

**Scheme 25:**
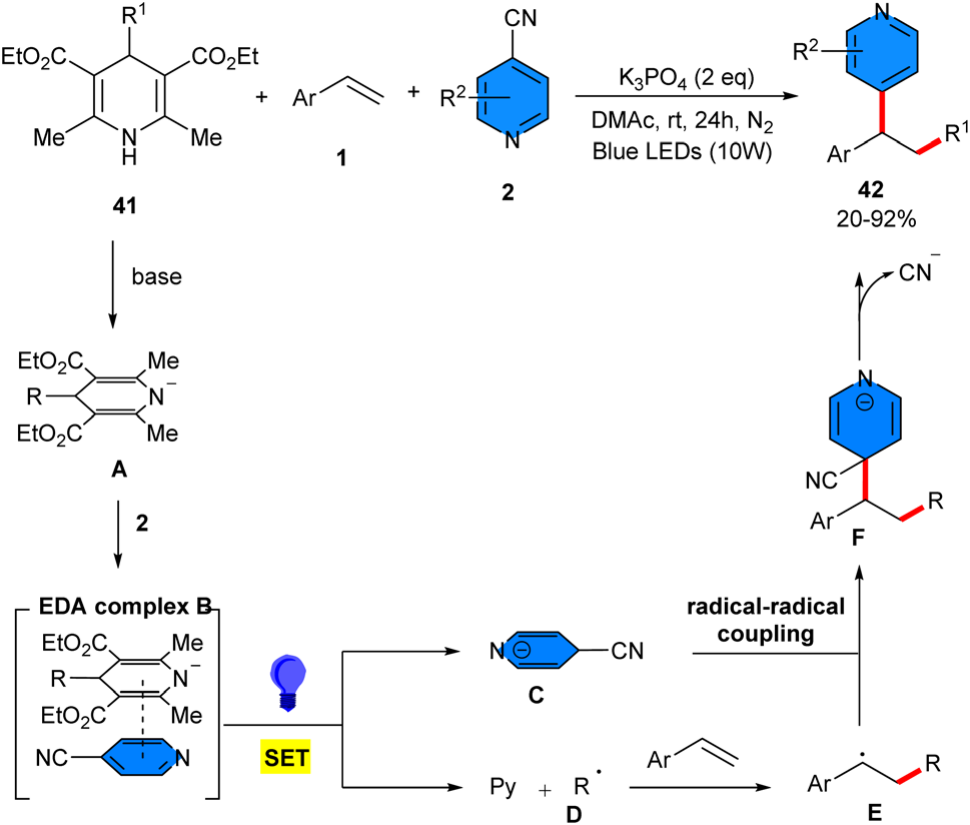
K_3_PO_4_-mediated alkylarylation of alkenes with 4-cyanopyridines and 4-cyclohexyl-DHP.

K_3_PO_4_-mediated 1,2-alkylheteroarylation of alkenes 1 was carried out by using 4-cyclohexyl-DHP 41 and 4-cyanopyridines 2 as alkylating and arylating reagents, respectively, under visible light triggered photosensitizer-free conditions (Scheme 25).^21^ The use of K_2_CO_3_ or DBU instead of K_3_PO_4_ can produce the target products in lower yields (24–36%), while Cs_2_CO_3_ resulted in good yield (84%). The base, light and inert atmosphere were all necessary, and the performance of the reaction in the presence of 425 nm or 480 nm led to 76% and 74% yield of the product, indicating the importance of set of LED irradiation at exactly 455 nm, 10 W. Mechanistic investigations, including UV-vis absorption spectroscopy and NMR titration experiments, suggested the formation of an EDA complex between 4-alkyl-DHPs and 4-cyanopyridines under basic conditions. Wherein, primary, secondary, and tertiary C(sp^3^)-centered radicals were generated by homolytic cleavage of 4-alkyl-DHPs through a SET process. According to the mechanism, first, base promoted deprotonation of 4-alkyl-DHPs 41, generating anion A, which then combined with 4-cyanopyridine to create the EDA complex B. This complex induced a SET from anion A to 4-cyanopyridine, resulting in pyridyl radical anion C and alkyl radical D. The latter added to styrene to obtain the nucleophilic benzylic radical E, which added to C, followed by intermolecular radical–radical coupling, towards intermediate F. Finally, the product 42 was delivered upon decyanation. The utility of this method was demonstrated by the gram-scale synthesis of the product (1.25 gr, 81% yield) and late-stage functionalization of medicinally important molecules.

**Scheme 26:**
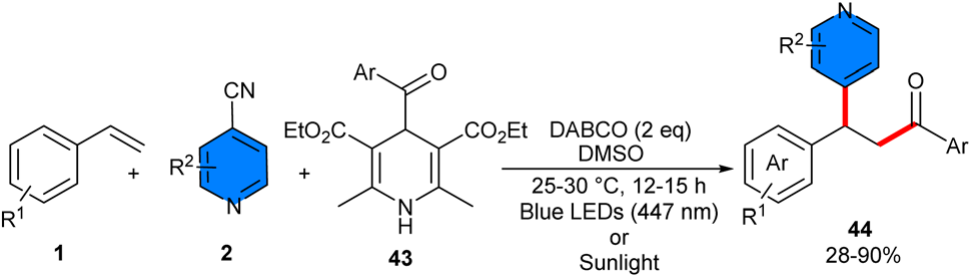
DABCO-mediated reaction of styrenes, 4-cyanopyridines, and 4-acyl-1,4-dihydropyridines.

**Scheme 27:**
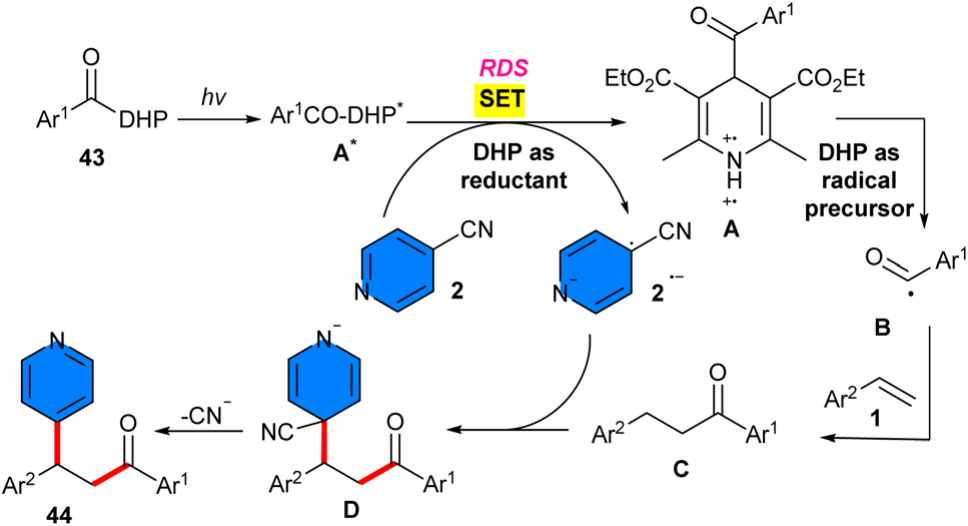
Possible mechanism for DABCO-mediated reaction of styrenes, 4-cyanopyridines, and 4-acyl-1,4-dihydropyridines.

In 2024, a wide spectrum of β-pyridinyl ketone compounds were synthesized through photo-induced acylarylation of styrenes 1 with 4-acyl-1,4-dihydropyridines (DHPs) 43 and 4-cyanopyridines 2 (Scheme 26).^41^ There was no need for any photocatalyst, and 4-acyl-1,4-DHP served as a photoreductant to reduce 4-cyanopyridine to its radical anion intermediate and as a radical precursor to generate the 4-acyl radical. To expand the scope of substrates, many substituted styrenes investigated, in which styrenes with halogen groups displayed slightly higher reactivity than styrenes with electron-donating groups. Styrenes with more sterically hindered *ortho-*substituent is also suitable for this transformation. Unlike styrenes, 4-benzoyl-1,4-DHPs with electron-donating groups on the phenyl ring exhibited high reactivity compared to electron-withdrawing groups. The reactivity of 4-cyanopyridines bearing different functional groups such as Me, Cl, Ph, and OMe at *ortho*- or *meta*-position had good tolerance. Radical trapping and radical clock experiments suggested a radical pathway including benzoyl radical intermediates. The on-off experiment confirmed the necessity of continuous light irradiation. Furthermore, the Stern–Volmer experiment showed that 4-cyanopyridine quenched the fluorescence generated by A*, indicating the SET process. To initiate the reaction process, light irradiation was used to convert acyl DHP 43 to acyl DHP A*. A single electron reduction between A* and 4-cyanopyridine 2 occurred to form radical cation A^+^˙ and radical anion B^−^˙. Then, A^+^˙ was cleaved, generating benzoyl radical, which was captured by styrene 1. The resulting benzyl radical C was further coupled with radical anion B^−^˙, affording intermediate D, which liberated cyanide to furnish the product 33 (Scheme 27). The gram-scale of the method could be performed under 447 nm blue LEDs (1.68 gr, 56%) and sunlight (0.60 gr, 40%).

**Scheme 28:**
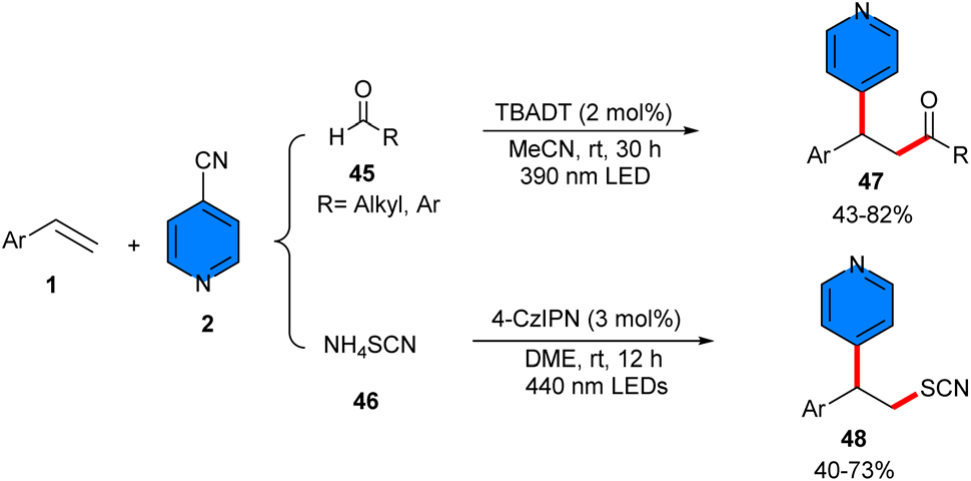
TBAD-catalyzed acylarylation and thiocyanoarylation of alkenes.

**Scheme 29:**
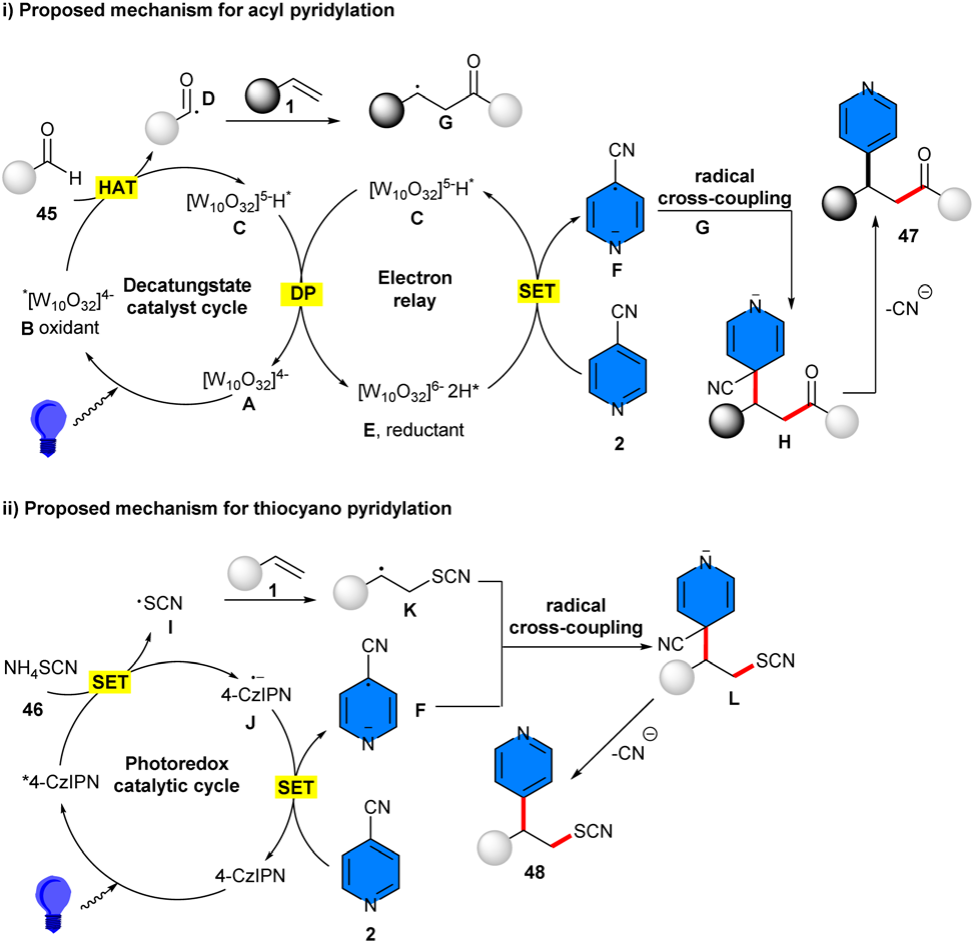
Possible mechanisms for TBAD-catalyzed acylarylation and thiocyanoarylation of alkenes.

In 2025, metal-free acylarylation and thiocyanoarylation of alkenes was reported by Zhang and co-workers (Scheme 28).^42^ For acylarylation, they employed aldehydes 45 and 4-cyanopyridines 2 as acylating and arylating agents, and the radical–radical coupling process was carried out in the presence of tetrabutylammonium decatungstate (TBADT) as a photocatalyst. Eliminating either TBAD or visible light completely prevented the reaction. None of the other photocatalysts PC1, PC2, and PC3, were workable. Substrate scope with respect to primary, secondary alkyl aldehydes, and aromatic aldehydes, as well as late-stage modification of bioorganic molecules, demonstrated the potential application of this method. The use of 4CzlPN as a photocatalyst could promote thiocyanoarylation of alkenes 1 with NH_4_SCN 56 and 4-cyanopyridine 2. Various β-pyridinyl thiocyanates were obtained smoothly under visible light conditions. Radical trapping with TEMPO and ally sulfone confirmed a radical pathway, involving visible light-induced formation of the decatungstate anion of A, followed by the conversion to the reactive excited state B. Then, B abstracted a hydrogen atom from the aldehyde, leading to the acyl radical D and singly reduced decatungstate C. The addition of D to alkene delivered the transient radical G. Disproportionation of C regenerated the active HAT photocatalyst A and doubly reduced decatungstate E. Subsequent single electron reduction of 4-cyanopyridine by E furnished the persistent radical anion F, which underwent radical–radical coupling with G to give intermediate H. Finally, the elimination of a cyanide delivered the target product 47 (Scheme 29i). For thiocyanoarylation, the excited-state photocatalyst PC* was reduced by NH_4_SCN, generating photocatalyst PC* J and the SCN radical I. The latter added to the alkene, forming the transient radical intermediate K. The single electron reduction of 4-cyanopyridine by J gave the persistent radical anion F, which coupled with radical K to afford intermediate M. The desired product 48 was obtained after the elimination of the CN group (Scheme 29ii).

**Scheme 30:**
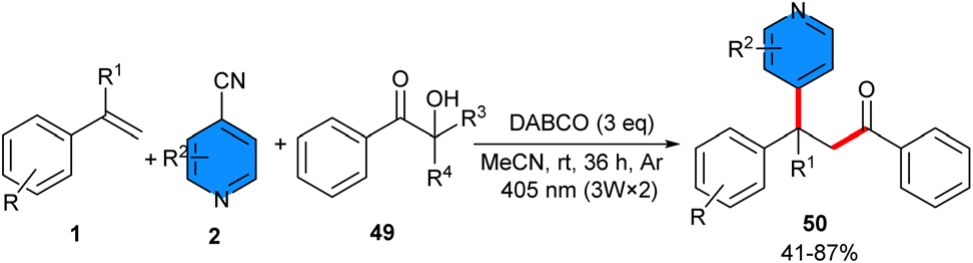
DABCO-mediated reaction of α-hydroxyketones, styrenes, and 4-cyanopyridine.

Visible light-induced DABCO-promoted three-component acylpyridylation of alkenes 1 with α-hydroxy ketones 49 and 4-cyanopyridines 2 was developed by Zeng and his team (Scheme 30).^43^ In this approach, α-hydroxy ketones played a triple role: (i) visible-light-activated photosensitizers, (ii) acylating reagents, and (iii) reductants. So, there was no need for a transition-metal catalyst, an external photosensitizer, or a reductive agent. None of the other bases, including CsF, NaH, KOMe, or DBU were as effective as DABCO in this reaction. Although Et_3_N led to the desired product in moderate yield (58%). The reaction did not proceed without 405 nm irradiation, or with 425/465 nm irradiation, which showcased the importance of the wavelength of *hv* in this strategy. Radical capturing experiments using TEMPO and 1,1-diphenylethylene dramatically diminished the reaction, and the radical clock reaction of vinyl cyclopropane with 4-cyanopyridine and α-hydroxy ketone resulted in an addition/ring opening product. These results supported a radical mechanism. The scalability of the synthetic method was shown by expanding substituted 4-cyanopyridines bearing different functional groups such as Me, *t*-Bu, OMe, Cl, CF_3_, Ph, 4-MePh, 4-OMePh, or 4-CO_2_MePh, whether located at the 2- or 3-position of the pyridine ring, which were well tolerated, and afforded the desired products in high efficiency and excellent regioselectivity. In the case of alkenes, high yields of products were obtained regardless of the position of substituents or types of functional groups. Notably, the exposed –OH, –CO_2_H, and –B(OH)_2_ groups was well retained in this system.

### 2.2. C–C and C–N bond formation

#### 2.2.1. Transition metal catalysis

**Scheme 31:**
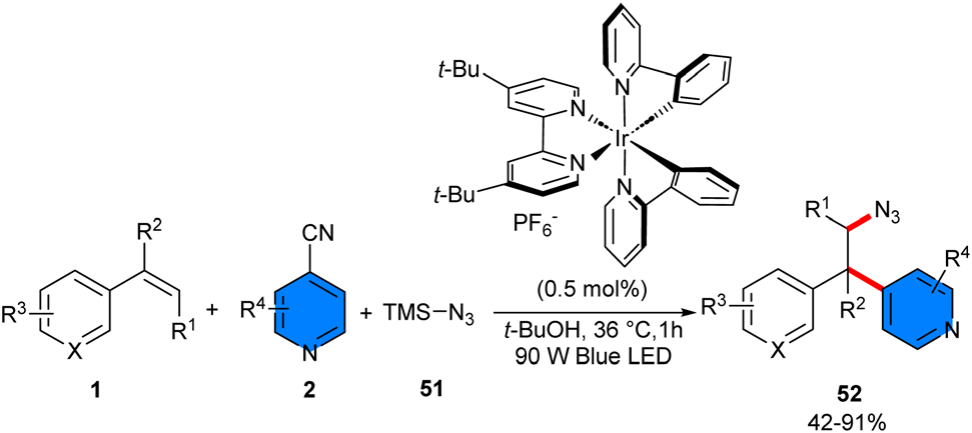
Ir-catalyzed reaction of alkenes, 4-cyanopyridines, and TMSN_3_.

**Scheme 32:**
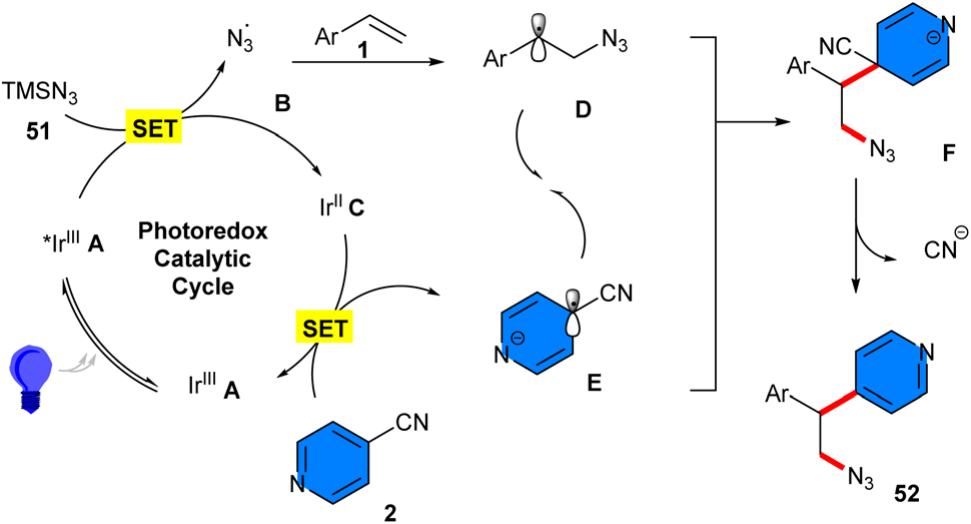
Possible mechanism for Ir-catalyzed reaction of alkenes, 4-cyanopyridines, and TMSN_3_.

In 2019, Chu and co-workers reported a three-component reaction between alkenes 1, 4-cyanopyridines 2, and TMSN_3_51 (Scheme 31).^44^ Among the investigation of various photocatalysts, it was found that only Ir[dF(CF_3_)ppy]_2_(dtbbpy)PF_6_, and 4CzlPN were suitable for this azidoarylation, and none of the other catalysts; Ir(ppy)_3_, Ru(bpy)_3_Cl_2_·6H_2_O, Ru(phen)_3_Cl_2_·6H_2_O, and Eosin Y, were workable. Various styrene derivatives bearing both electron-donating and electron-withdrawing groups at the aryl ring tolerated well in this redox–neutral reaction with pyridines containing substituents, such as alkyl, aryl, fluoro, chloro, and cyano. However, the reaction was affected by the steric hindrance at the *ortho*-position of 4-cyanopyridine, where 3-methylisonicotinonitrile showed moderate reactivity. A plausible mechanism for this transformation was suggested. Firstly, Ir(ppy)_2_(dtbbpy)PF_6_ was converted to the photoexcited state Ir* A under visible light irradiation, followed by engaging in a single-electron transfer (SET) with azide to produce the electrophilic azido radical B. Then, the azido radical B added to styrene, leading to benzylic radical D. On the other hand, a single-electron reduction occurred between Ir(ii) B and cyanopyridine 2, generating the pyridyl radical anion E. Afterwards, E reacted with the benzylic radical D, providing intermediate F, which released a CN anion to yield the product 52 (Scheme 32). To demonstrate the synthetic utility of this method, the authors converted β-azido pyridine into β-amino pyridine, β-amido pyridine, and β-azido heterocycles.

### 2.3. C–C and C–S bond formation

#### 2.3.1. Transition metal catalysis

**Scheme 33:**
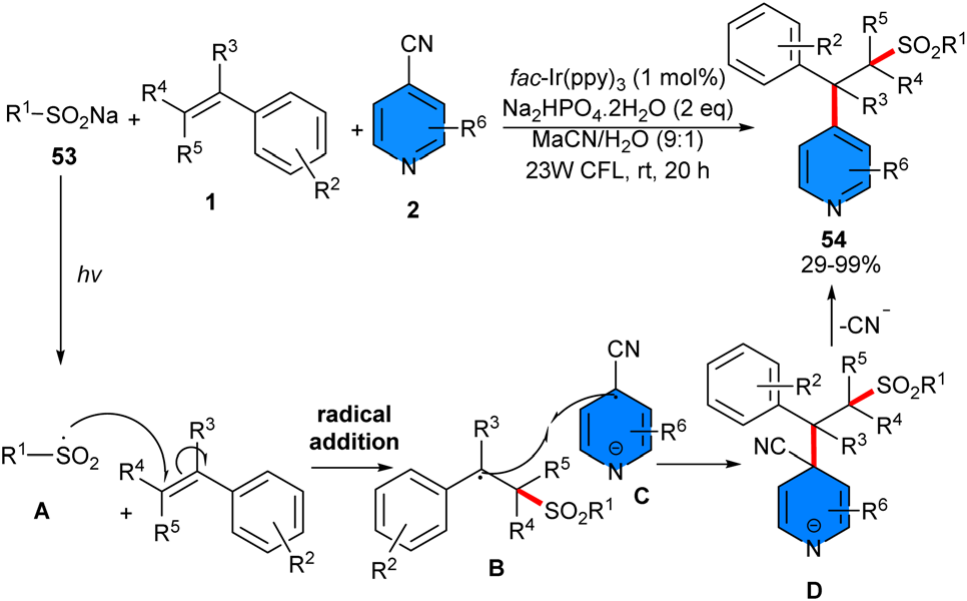
Ir-catalyzed reaction of styrenes, 4-cyanopyridines and sodium sulfinates.

In 2019, Opatz's team developed a three-component photoredox reaction including styrenes 1, 4-cyanopyridines 2, and sodium sulfinates 53 (Scheme 33).^45^ Only 1 mol% of *fac*-Ir(ppy)_3_ can efficiently catalyze sulfonylation/arylation of styrenes under visible light irradiation. Styrenes bearing electron-donating and electron-withdrawing groups, all displayed significant results (80–99% yields). 4-Cyanopyridines bearing a strongly electron-donating OMe group gave a moderate yield (35%), which is likely due to the lower stability of the corresponding radical anion or to less favorable kinetics of the SET step. For linear alkyl sulfonates, steric hindrance had a major impact on the reaction. A sequence of radical formation and radical combination was proposed for this synthetic method, where persistent radical anions C of the reduction of 4-cyanopyridine did not homodimerize due to electrostatic repulsion and instead selectively trapped the benzylic radical B generated from the addition of electrophilic sulfonyl radicals A to the styrene 1. The product formation was completed by the liberation of cyanide from D. It should be noted that both persistent radical anions and electrophilic sulfonyl radicals were formed under the photoredox iridium catalytic cycle.

#### 2.3.2. Organocatalysis

**Scheme 34:**
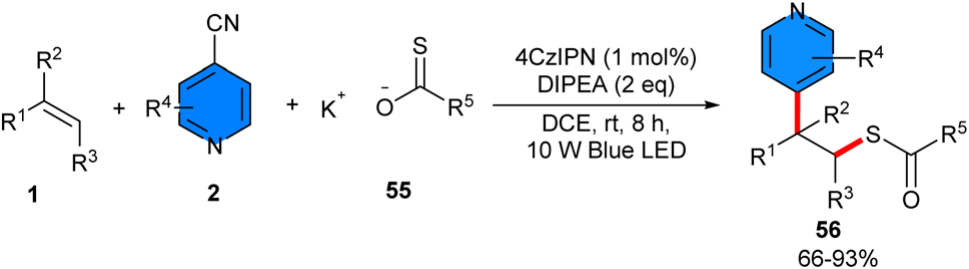
4CzlPN-catalyzed reaction of aryl cyclopropanes, 4-cyanopyridines, and thioacetates or thiosulfonates.

**Scheme 35:**
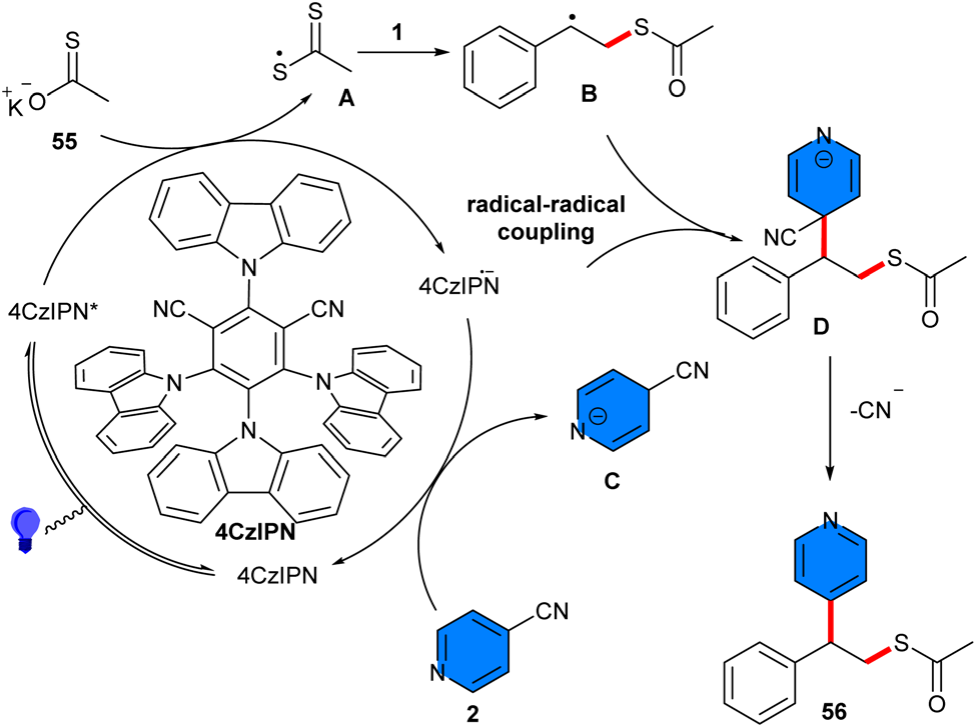
Rational mechanism for 4CzlPN-catalyzed reaction of aryl cyclopropanes, 4-cyanopyridines, and thioacetates or thiosulfonates.

Thiolation/pyridinylation of alkenes was achieved under visible light-induced reaction of styrenes 1, 4-cyanopyridines 2, and thioacetates or thiosulfonates 55 (Scheme 34).^46^ By employing 4CzIPN as a photocatalyst and DIPEA as a base, a wide array of 1,2-pyridylthiolation products were synthesized in high to excellent yields with high regioselectivity. Notably, other photocatalysts, including Eosin Y, Rose Bengal, and *fac*-Ir(ppy)_3_, also gave the target product in acceptable yields (63–78%). The absence of 4CzIPN or light resulted in trace yield, and the reaction without DIPEA gave 36% yield. The reaction was found to be not influenced by the electronic or steric effects. Several 4-cyanopyridines with substituents at the *ortho*- or *meta*-position were well tolerated with alkenes bearing different functional groups on the *ortho*-, *meta*- or *para*-position of the aryl ring, accessing the corresponding products in high yields. The possible mechanism started with the conversion of 4CzIPN to its excited state, followed by SET with compound 55 to generate a sulfur radical A. Then, A combined with alkene 1, producing the radical B. On the other hand, a single electron reduction between 4CzIPN·– and 4-cyanopyridine 2 led to the pyridyl radical anion C and regenerated the ground-state 4CzIPN to fulfill the photocatalytic cycle. Subsequent intermolecular radical–radical coupling between B and C forged intermediate D, which underwent aromatization *via* elimination of a cyanide anion, furnishing the product 56 (Scheme 35). The 1,2-pyridylthiolation products con also be converted to 4-(1-phenylvinyl)pyridine or dithioperoxoate structures. The gram-scale synthesis of two derivatives of 1,2-difunctionalized compounds was accomplished, giving 0.604 gr, 47% yield, and 1.089, 59% yield. Furthermore, the *in vitro* antitumor activities of some of the obtained products were screened and showed good antitumor activity.

**Scheme 36:**
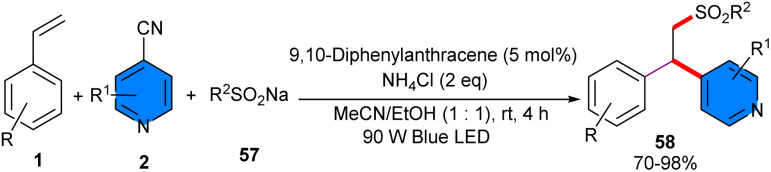
Photo-induced DPA-catalyzed reaction of alkenes, 4-cyanopyridines, and sodium sulfinates.

An organic photoredox catalysis system was utilized for three-component reaction involving alkenes 1, 4-cyanopyridines 2, and sodium sulfinates 57 (Scheme 36).^47^ 9,10-Diphenylanthracene (DPA) served as a photocatalyst to promote sulfonylative pyridylation of alkenes, leading to β-pyridyl sulfone derivatives in good to excellent yields (69–98%). Besides, eosin Y could produce the desired product in 82%, while other catalysts, such as 4CzIPN, benzophenone, and anthracenes with 9,10-CN or -OMe substituents, showed dramatically decreased efficiency (5–45% yield). In general, the reaction was not significantly influenced by the electronic and steric properties of substrates. Replacing 4-cyanopyridine with 1-cyano-isoquinoline or unprotected azaindole nitrile could also regioselectively produce the corresponding products in high yields (84% and 74%, respectively). The synthesis of synthetic complex drug-derived molecules in 60–97% yields, and the gram-scale preparation of the product in 4 mmol, 89% yield, confirmed the synthetic application of this metal-free method.

**Scheme 37:**
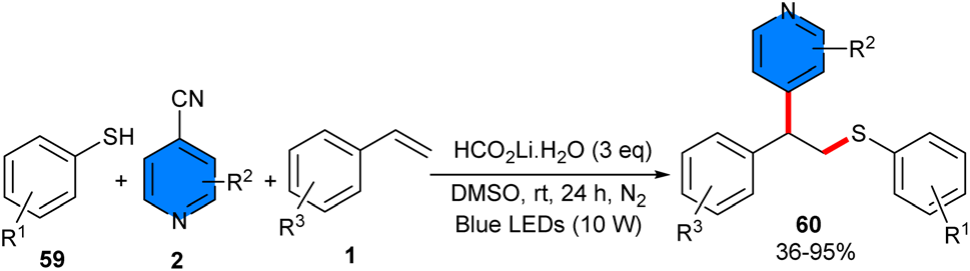
4CzIPN-catalyzed reaction of alkenes, 4-cyanopyridines, and thiophenes.

**Scheme 38:**
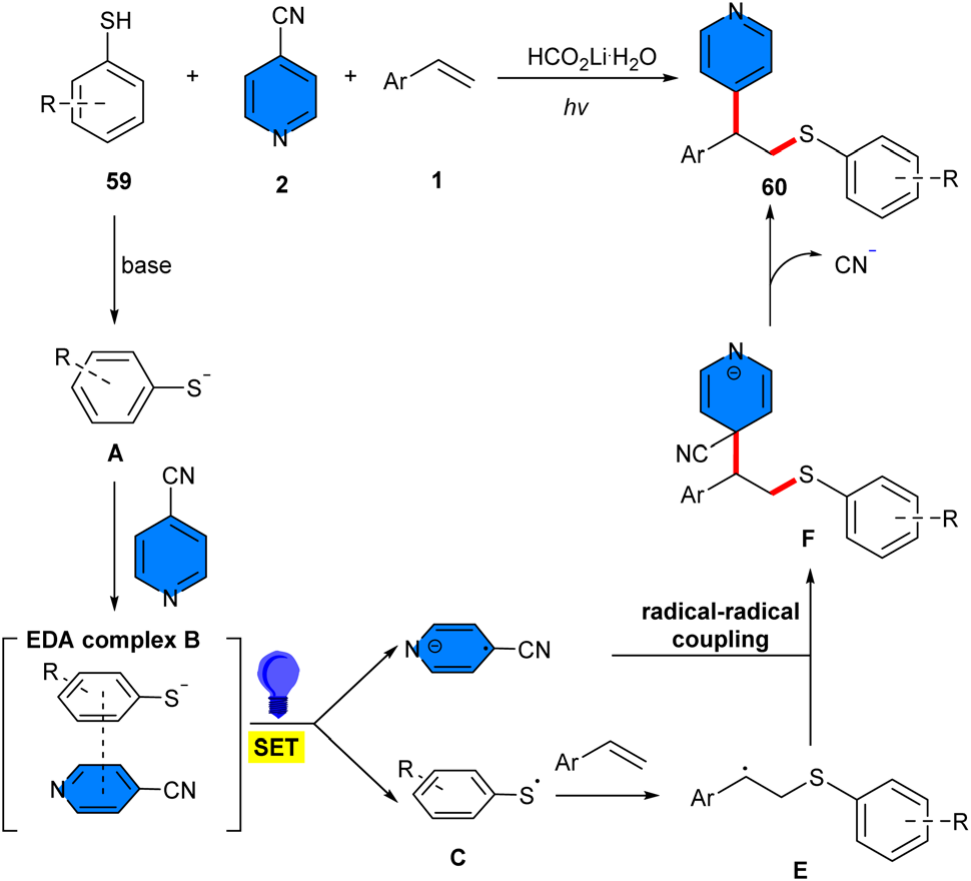
Possible mechanism for metal-free reaction of alkenes, 4-cyanopyridines, and thiophenes.

Thiolation/pyridylation of styrenes 1 occurred using 4-cyanopyridines 2 and thiophenes 59 under metal-free conditions (Scheme 37).^48^ Diverse styrenes bearing either electron-donating or electron-withdrawing groups on the phenyl moiety were suitable for this reaction, giving the products in moderate to good yields. 4-Cyanopyridines bearing electron-donating groups at the 2- or 3-position were well tolerated (41–95% yields). While 4-cyanopyridines with halogen groups gave a complex mixture of products. For thiophenols, both electron-rich and electron-poor substrates are considered at different positions of the aryl ring. Based on the radical trapping and radical clock experiments, a radical mechanism was suggested, in which HCO_2_Li·H_2_O assisted deprotonation of thiophenol 59, leading to the thiolate anion A. An EDA complex B was generated between the thiolate anion and 4-cyanopyridine, which was confirmed by UV-vis spectroscopy. Subsequently, under visible-light irradiation, the EDA complex B underwent a SET from the thiolate anion to 4-cyanopyridine, leading to the thiyl radical C and pyridyl radical anion D. Then, C added to styrene 1 to obtain the nucleophilic benzylic radical E, which moved through intermolecular radical–radical coupling with D to afford intermediate F, provided the product 60 by eliminating a cyanide anion (Scheme 38).

### 2.4. C–C and C–O bond formation

#### 2.4.1. Transition metal catalysis

**Scheme 39:**
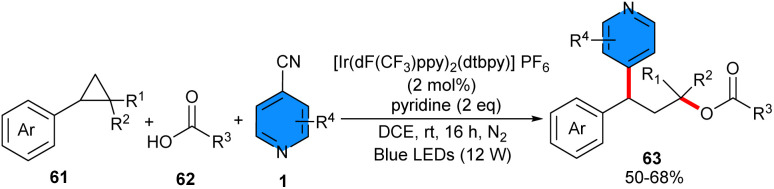
Ir-catalzed reaction of aryl cyclopropanes, 4-cyanopyridines, and carboxylic acids.

**Scheme 40:**
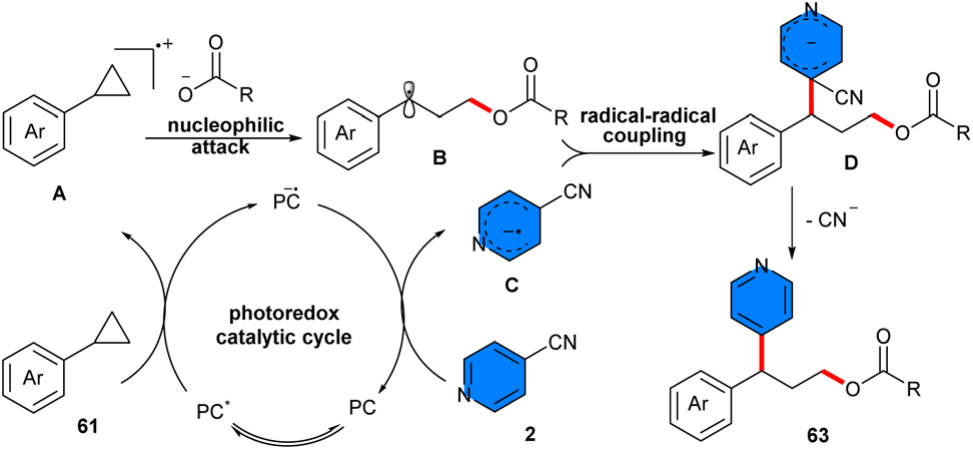
Catalytic cycle for Ir-cata;yzed reaction of aryl cyclopropanes, 4-cyanopyridines, and carboxylic acids.

**Scheme 41:**
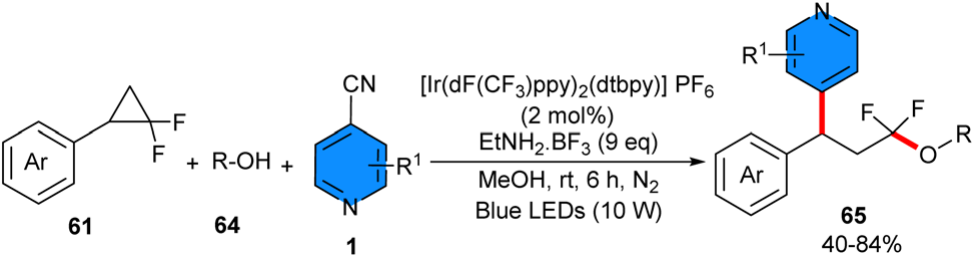
Ir-catalzed reaction of aryl cyclopropanes, 4-cyanopyridines, and alcohols.

In 2024, 4-cyanopyridines 2 and carboxylic acids 62 were used as coupling partners for 1,3-oxypyridylation of aryl cyclopropanes 61 in the presence of 2 mol% of Ir[dF(CF_3_)-ppy]_2_(dtbpy)PF_6_ as a photocatalyst and pyridine as a base under irradiation with 12 W blue LEDs (Scheme 39).^49^ Due to the alkene-like reactivity of cyclopropanes, these synthons could be underwent electrophilic activation with Lewis species. Thus, method involved photocatalytic ring opening and radical 1,3-difunctionalization of aryl cyclopropanes. The scope of substrates respect to 4-cyanopyridines with various substituents at C2 or C3 position, such as aryl, alkynyl, cyano, fluoro, and alkyl groups, were well tolerated. Interestingly, 4-cyanoquinoline also participated well in furnishing moderate yield of product (53%). Furthermore, a wide range of benzoic acids and heteroaromatic benzoic acids featuring different electronic properties regardless of steric hindrance, as well as aliphatic acids all showed good compatibility in this transformation. In order to perform a large-scale reaction, 1 mmol of each substrate was used, leading to a 67% isolated yield. According to the results of the radical-trapping experiment, a radical route was proposed for this process, involving a SET between the excited-state photocatalyst and aryl cyclopropane 61, leading to an arylcyclopropyl radical cation intermediate A and the radical anion of the photocatalyst. Subsequently, nucleophilic attack of the carboxylate ion on the electron-deficient intermediate induced ring opening of cyclopropane, affording benzylic radical intermediate B. Then, the single-electron reduction of 4-cyanopyridine 2 by the reduced photocatalyst rendered pyridyl anion intermediate C and regenerated the ground-state photocatalyst, to complete the photocatalytic cycle. Sequential radical–radical coupling of the benzylic radical with pyridyl radical anion intermediates, followed by decyanation/aromatization produced the target product 65 (Scheme 40). Instead of carboxylic acids, alcohols could be used as coupling partners for 1,3-alkoxypyridylation of aryl cyclopropanes (Scheme 41).^50^ In this regard, *gem*-difluorinated cyclopropanes, 4-cyanopyrines, and a range of aliphatic and benzylic alcohols reacted in the presence of 2 mol% of Ir[dF(CF_3_)-ppy]_2_(dtbpy)PF_6_ under irradiation of 10 W blue LEDs at room temperature. The presence of two fluorine atoms at the cyclopropane ring can stabilize the generated cation and facilitate the subsequent nucleophilic attack by the alcohol and the following ring-opening step. The resulting benzylic radical smoothly underwent radical coupling with the persistent pyridyl radical anion to furnish the final coupling product concomitant with rapid decyanation/rearomatization. The gram-scale synthesis of the product (1.23 gr, 84%) and the late-stage functionalization of natural products and drugs demonstrated the synthetic application of this method.

### 2.5. C–C and C–Si bond formation

#### 2.5.1. Transition metal catalysis

**Scheme 42:**
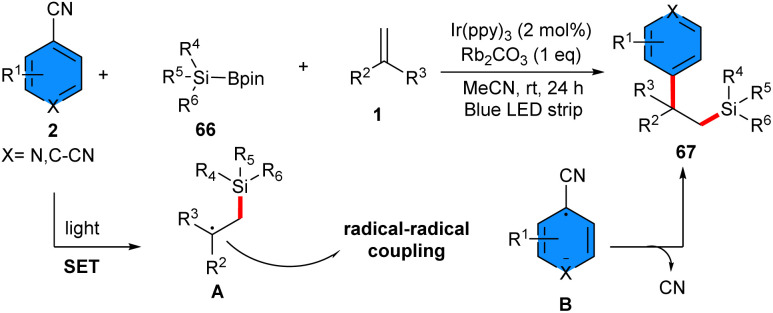
Ir-catalyzed reaction of alkenes, 4-cyanopyridines and silylboronates.

The synthesis of a series of silicon-containing 1,1-diaryl motifs 67*via* arylsilylation of aryl alkenes could be carried out by using 4-cyanopyridines 2 and silylboronates 66 (Scheme 42).^51^ Ir(ppy)_3_ as a photocatalyst, and Rb_2_CO_3_ as a base, efficiently facilitated SET reaction of base/silylboronate adducts and (hetero)aryl nitriles in the presence of irradiation of a blue LED strip, generating the silyl radicals and (hetero)aryl nitrile radical anions. These radicals underwent sequential coupling with alkenes, yielding valuable 1,1-diaryl compounds with a silicon group at the β-position. Styrenes bearing both electron-donating and electron-withdrawing functionalities at the different positions of the benzene ring, as well as 1,1-disubstituted alkenes containing substituents at the benzene ring (Bpin, ester, heterocycles, *etc.*), were all workable to this protocol. 4-Cyanopyridines bearing functionalities at the C2-(cyclopropyl, *t*Bu, Me, F) and C3-(MeO, Me, F, aryl, MeS, CO_2_Et, 2-thienyl) positions of the pyridine ring were all applicable. Additionally, 1-cyanoquinoline and 1,2-dicyanobenzene were also suitable in this coupling reaction. The practicality of this method was shown by the gram-scale synthesis of the product (1.3 gr, 78%), and diversification of some bioactive molecules.

#### 2.5.2. Organocatalysis

**Scheme 43:**
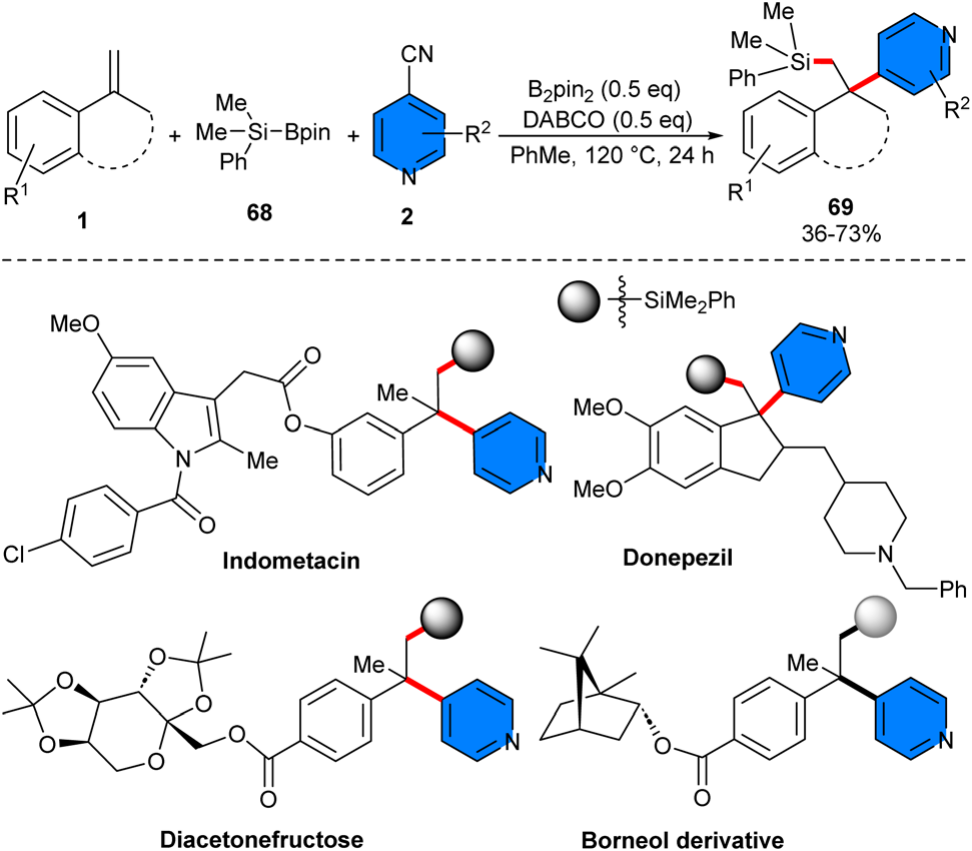
Metal-free reaction of alkenes, 4-cyanopyridines, and silyl boronate.

4-Cyanopyridine 2 and silyl boronate 68 can efficiently participate in 1,2-silylpyridylation of styrenes 1 under metal-free conditions (Scheme 43).^52^ To identify the mechanism, DFT calculation, radical trapping experiment, and radical click experiment were performed, in which it was found that 4-cyanopyridine plays dual functions; (i) the homolytic cleavage of the B–Si bond access to the silyl radical and the pyridine-boryl radical, (ii) the homolytic cleavage of the B–B bond in B_2_pin_2_ to generate pyridine-boryl radical for radical–radical coupling step. The gram-scale reaction provided a 61% yield of the product, and the silyl group could be converted to a hydroxyl group in 72% yield. Furthermore, late-stage modification of several drugs and natural products was performed in this work.

#### 2.5.3. Organocatalysis

**Scheme 44:**
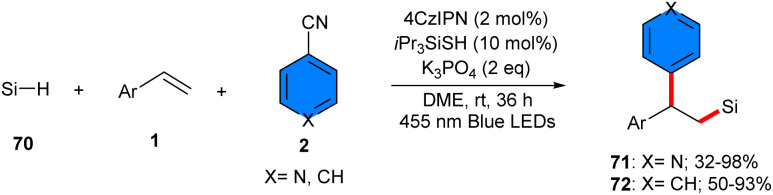
Metal-free reaction of alkenes, 4-cyanopyridines, and hydrosilanes.

**Scheme 45:**
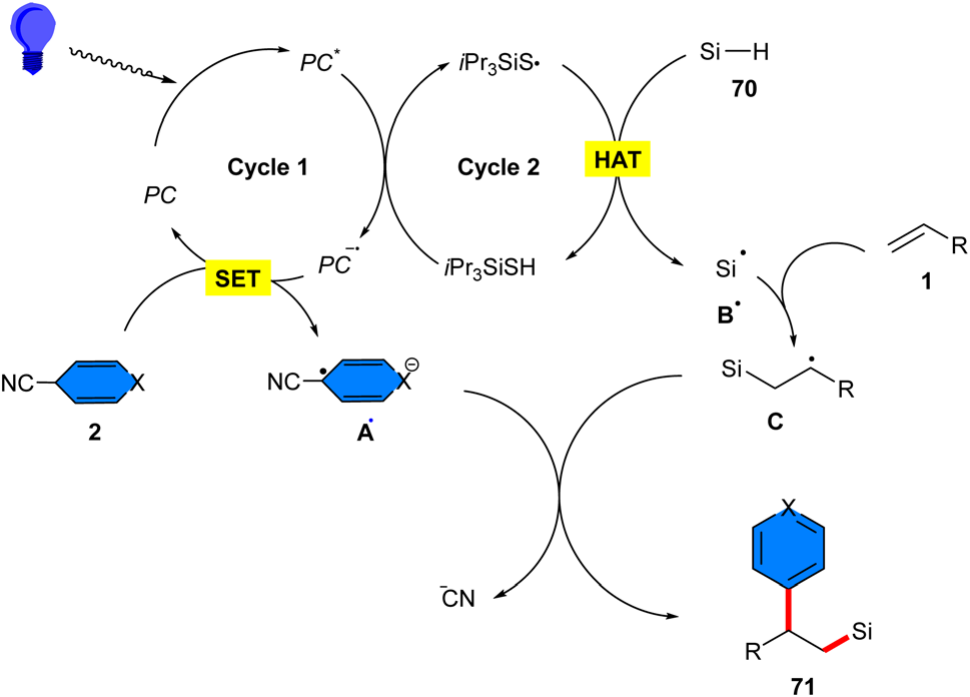
Possible mechanisms for photo-induced reaction of alkenes, 4-cyanopyridines, and hydrosilanes.

Arylsilylation of styrenes 1 with 4-cyanopyridines 2 and hydrosilanes 70 can be carried out under visible light conditions (Scheme 44).^24^ The reaction was performed either with the combination of 4CzIPN as a photocatalyst and triisoproylsilylthiol (*i*Pr_3_SiSH) as a HAT catalyst, or only using the HAT catalyst. Both methods yield moderate to high silicon-containing compounds. The organocatalysis cycle involved light-mediated excitation of 4CzIPN, followed by quenching by *i*Pr_3_SiSH in the presence of base. Then, the excited 4CzIPN* oxidized the HAT catalyst *i*Pr_3_SiSH to obtain the electrophilic radical *i*Pr_3_SiS˙, which abstracted an H-atom from hydrosilane 70 to form a silyl radical. Upon Giese addition to the C=C bond, the β-silylalkyl radical was formed, which coupled with the radical A˙, generated from the reduction of arylnitrile 2 by 4CzIPN^−^˙. The removal of the nitrile anion liberated the final product 71. For photocatalyst-free reaction system, it was anticipated that the EDA complex was excited by visible light to form radical A˙ and *i*Pr_3_SiS˙. Sequentially, the abstraction of hydrosilane towards the silyl radical, followed by Giese addition access to β-silylalkyl radicals, and coupling step yielded the final product (Scheme 45).

### 2.6. C–C and C–P bond formation

#### 2.6.1. Organocatalysis

**Scheme 46:**
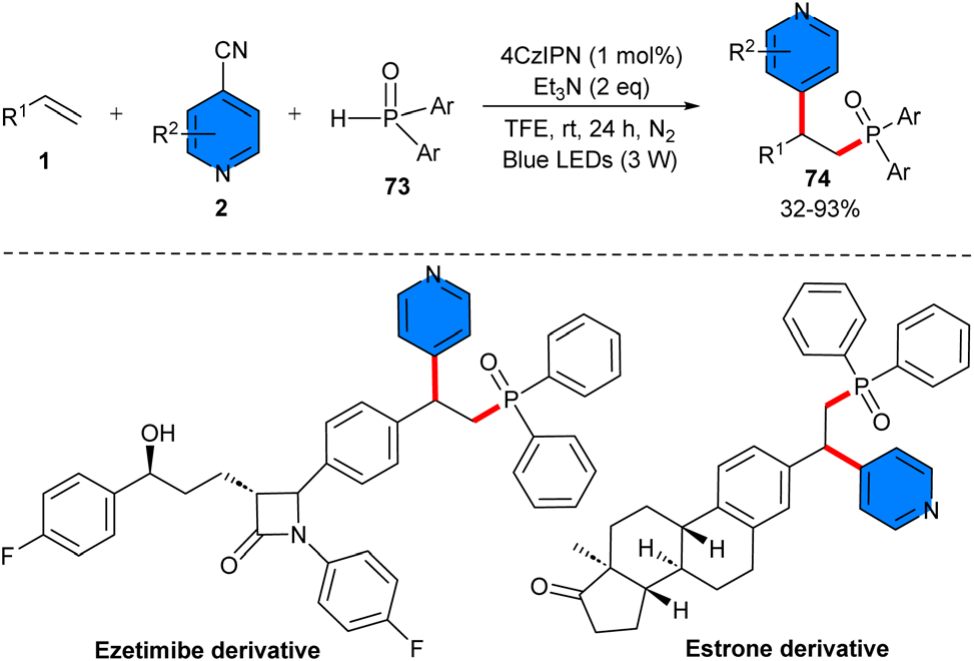
4CzIPN-induced reaction of styrenes, 4-cyanopyridines, and diphenylphosphine oxide.

**Scheme 47:**
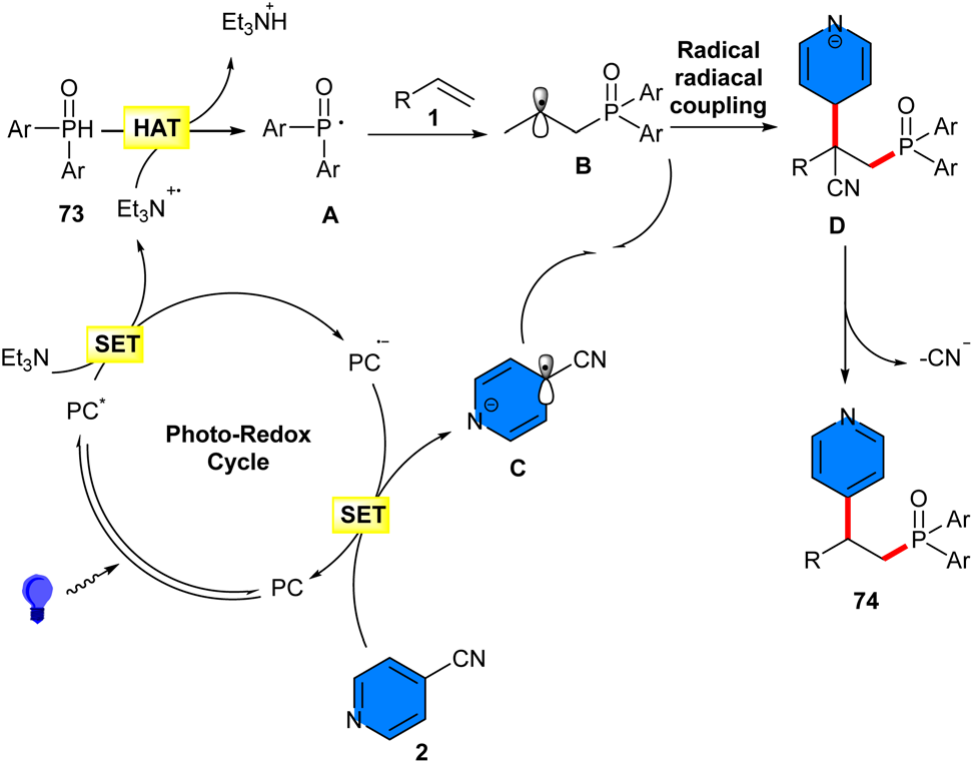
Possible mechanism for 4CzIPN-induced reaction of styrenes, 4-cyanopyridines, and diphenylphosphine oxide.

In 2021, phosphinoylation pyridylation of alkenes 1 using 4-cyanopyridines 2 and diphenylphosphine oxides 73 was reported by Shen *et al.* (Scheme 46).^53^ Screening of various organophotocatalysts, including 4CzIPN, fluorescein, eosin Y, Mes^−^Acr^+^, showed that the best result could be obtained using 4CzIPN as a photoredox catalyst. The synthetic value of this protocol was shown by the gram-scale synthesis of the product (1.44 gr, 75% yield), as well as the modification of some natural products. Based on the results of radical inhibition and radical clock experiments, a radical mechanism was proposed for this transformation, which started with the oxidation of 4CzIPN by visible light, generating 4CzIPN*, which then oxidized Et_3_N *via* a SET to produce Et_3_N˙^+^ and PC˙^–^. Then, hydrogen atom transfer (HAT) of diarylphosphine oxide 73 with Et_3_N˙^+^ provided Et_3_NH^+^ and phosphinoyl radical I. The latter was added to alkene 1, forming radical B. Upon single-electron reduction of 4-cyanopyridine by the reduced PC˙^–^, the persistent radical anion C and PC were generated. Subsequently, radical–radical coupling of the transient radical B and the persistent radical anion C delivered intermediate D, followed by rearomatization to afford product 74 (Scheme 47).

**Scheme 48:**
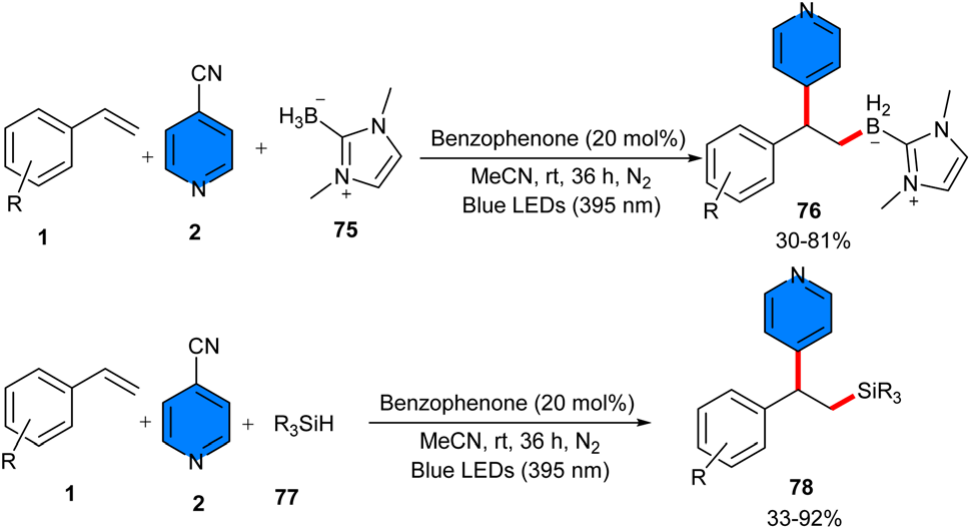
Benzophenone-catalyzed reaction of alkenes, 4-cyanopyridines, and NHC–BH_3_ complexes, or hydrosilanes.

In 2025, Liu and Zhou were able to accomplish borylative/silylative pyridylation of vinylarenes 1 under metal-free photocatalytic conditions (Scheme 48).^54^ The three-component reaction of vinylarenes, 4-cyanopyridine 1, and NHC–BH_3_ complexes 75 or hydrosilanes 77 leading to the synthesis of β-pyridinyl boranes 76 or β-pyridinyl silanes 78, respectively. Silylarylation and borylarylation of alkenes resulted in moderate to high product yields. In both coupling reactions, styrenes featuring electron-donating groups showed lower reactivities than those with halogen and electron-withdrawing substituents. Several mechanistic investigations, including radical quenching reaction with TEMPO, radical clock experiment with vinylcyclopropane, and light on/off experiment, suggested the involvement of a radical pathway including benzyl radicals. KIE experiment for borane (*B*_H_/*B*_D_ = 1.0) indicated that the breaking of the B–H bond may not be the rate-limiting step. Furthermore, the method presented the late-stage functionalization of pharmaceutically valuable molecules with good to excellent yields.

## 3. Conclusions

As shown in this review, 4-cyanopyridine derivatives could easily participate in 1,2-difunctionalization of alkenes, including alkylpyridylation, arylpyridylation, carbopyridylation, azidopyridylation, pyridylboration, pyridylphosphinoylation, pyridylsilylation, and pyridylsolfonylation. The reactions proceeded in the presence of transition metal or non-metal photocatalysts under visible light irradiation. All transformations were carried out under mild conditions, although with a long reaction time. Notably, both metal-catalyzed reaction, and metal-free reactions afforded acceptable product yields. As discussed in mechanisms, the formation of the cyanopyridine radical anion was the same in all reactions. Furthermore, the radical species generated from the second substrate was trapped by styrene to form a benzyl radical, and radical–radical coupling proceeded with the cyanopyridine radical species.

Despite various impressive efforts in the synthesis of pyridine-containing compounds, and due to the structural modification of the pyridine core, especially in the late-stage functionalization of drug candidates, it is still highly required to develop atom-economic and green strategies for the synthesis and functionalization of pyridine scaffolds.

For instance, replacing toxic transition metal catalysts with organocatalysts, NHCs, and bases could be highly desirable. Merging electrochemistry with photochemistry to constitute an efficient and sustainable reaction system is also desirable.

## Conflicts of interest

There are no conflicts to declare.

## Data Availability

All data associated with this manuscript are available within the referenced articles.
